# Aberrant promoter hypermethylation of miR-335 and miR-145 is involved in breast cancer PD-L1 overexpression

**DOI:** 10.1038/s41598-023-27415-8

**Published:** 2023-01-18

**Authors:** Sara Hajibabaei, Fattah Sotoodehnejadnematalahi, Nahid Nafissi, Sirous Zeinali, Masoumeh Azizi

**Affiliations:** 1 Department of Biology, School of Basic Science, Science and Research Branch, Islamic Azad University, Tehran, Iran; 2 Breast Surgery Department, Iran University of Medical Sciences, Tehran, Iran; 3 Molecular Medicine Department, Biotechnology Research Center, Pasteur Institute of Iran, 69th Pasteur Street, Kargar Avenue, Tehran, Iran

**Keywords:** Biotechnology, Cancer, Cell biology, Drug discovery, Genetics, Immunology, Molecular biology, Biomarkers, Diseases, Medical research, Molecular medicine, Oncology

## Abstract

PD-L1 is one of the most important immune checkpoint molecules in breast cancer that plays an important role in suppressing the immune system when confronted with tumor cells and is regulated by various microRNAs. Among them, microRNA-335-3p and microRNA-145-5p, regulated by DNA methylation, have tumor suppressor activities. We studied the role of miR-335 and -145 on PD-L1 suppression in breast cancer. The expression of miR-355 and miR-145 was significantly downregulated in BC tissues and cell lines compared to their controls, and their downregulation was negatively correlated with PD‐L1 overexpression. In-silico and luciferase reporter systems confirmed that miR-335 and -145 target PD-L1. In BC tissues and cell lines, cancer-specific methylation was found in CpG-rich areas upstream of miR-335 and-145, and up-regulation of PD-L1 expression was connected with hypermethylation (r = 0.4089, P = 0.0147, and r = 0.3373, P = 0.0475, respectively). The higher levels of miR-355 and -145 in BC cells induced apoptosis, arrested the cell cycle, and reduced proliferation significantly. In summary, we found that miR-335 and -145 are novel tumor suppressors inactivated in BC, and these miRs may serve as potential therapeutic targets for breast cancer treatment.

## Introduction

Breast cancer (BC) is the most common cancer among women worldwide, as in 2021^1^, according to the American Cancer Society, an estimated 281,550 women are expected to be diagnosed with breast cancer, and 43,600 women in the US are predicted to die due to breast cancer. While an increasing number of patients can be treated with surgery, radiation, and systemic therapies, a sizable minority are resistant to systemic therapy^2,3^.

In recent years, the treatment of BC has made significant progress, leading to the development of targeted treatment^2–4^. One of the most likely candidates for tumor immune-microenvironment in the incidence of BC is the change of PD-L1 expression^5^. The interaction of programmed cell death 1 (PD-1) receptor-ligand (PD-L1 or PD-L2) by tumors was highlighted as a significant inhibitory pathway to suppress immune control and inhibit T-cell activity^6–9^. PD-L1 overexpression in BC disrupts the immune system, making it one of the most promising immunotherapy targets for BC^10^. Additionally, PD-L1 molecules on tumor cells can predict treatment response and survival prognosis.

Breast cancer is highly heterogeneous at the molecular and clinical level^11^. The molecular characterization of BC cells demonstrates epigenetic changes, such as miRs' aberrant expression, that silence many genes. MiRNA dysregulation may contribute to the pathogenesis of BC^12–14^. miRs can function as tumor suppressors or oncogenes by regulating biological processes such as cell differentiation, proliferation, and apoptosis^15,16^. In breast cancer, miRNA expression is associated with clinical and molecular subtypes^17^, progression^18^, prognosis^19^, and oncogene expression^20^. The current data show that the restoration of miR-424-5p, miR-138-5p, miR-570-3p, miR-200c-3p, miR-383-5p, miR-34a-5p, miR-3609, miR-195-5p, and miR-497-5p can inhibit tumoral PD-L1 expression, transform an immunosuppressive tumor microenvironment into a pro-inflammatory one, suppress tumor proliferation, inhibit tumor migration, improve chemosensitivity, enhance tumor apoptosis, imprison the cell cycle, and repress the clonogenicity of TNBC cells^21^. DNA methylation is a significant epigenetic marker in mammals that can deregulate miR in cancer. Breast pre-invasive lesions have CpG island methylation alterations^22^. Furthermore, increasing evidence shows that some tumor-suppressive miRs are also epigenetically silenced by promoter DNA methylation in cancers^23–25^, suggesting these miRs' diagnostic and therapeutic potential and showing that abnormal miR expressions contribute to human cancers^26^.

The miR network controls PD-L1 and PD-1 levels, regulating immune checkpoint-related genes through a complex mechanism^27–29^. A recent study showed that the miR-34 family is involved in regulating PD-L1 expression by targeting the 3'UTR of PD-L1. They suggested that using miR with standard treatment might represent a new approach in cancer treatment^30^. Tavazoie et al.^13^ reported miR-335 as a metastasis suppressor gene. Furthermore, they discovered that restoring miR-335 expression in malignant BC cells can prevent metastasis to the lung and bone. The findings show that miR-145 also regulates the expression of genes that have important functions in the invasion, migration, and metastasis of cancer cells. The expression of miR-145 as a tumor suppressor is reduced in various cancer cell lines in solid tumors, including breast cancer^31^.

It is proposed that in tumor cells, PD-L1 is up-regulated in response to excessive immune reactions and that they adopt this mechanism to evade tumor immunity^32^. We hypothesized that downregulation of miR-335 and-145 in BC cells promoted proliferation, cell cycle, and inhibition of apoptosis through overexpression of PD-L1, mediated via promoter methylation of miR-335 and -145, suggesting that miR-335 and -145 might have potential as novel therapeutic targets for BC.

The present study aimed to investigate the clinical significance and prognostic value of miR-335 and miR-145 in BC. The results indicated that PD-L1 was the direct target of miR-335 and that miR-145 acted as a tumor-suppressor gene in BC. We found CpG-rich regions upstream of the miR-335 and -145 genes. Therefore, the purpose of the present study was to assess the expression of miR-335 and -145 with the ability to regulate PD-L1 expression in BC tissue and cell lines, as well as determine the association between these factors and the rate of methylation in the promoter of miR-335 and-145 in BC tumors and cell lines.

## Results

### PD-L1 expression is upregulated in BC tissues and cell lines

**Figure 1:**
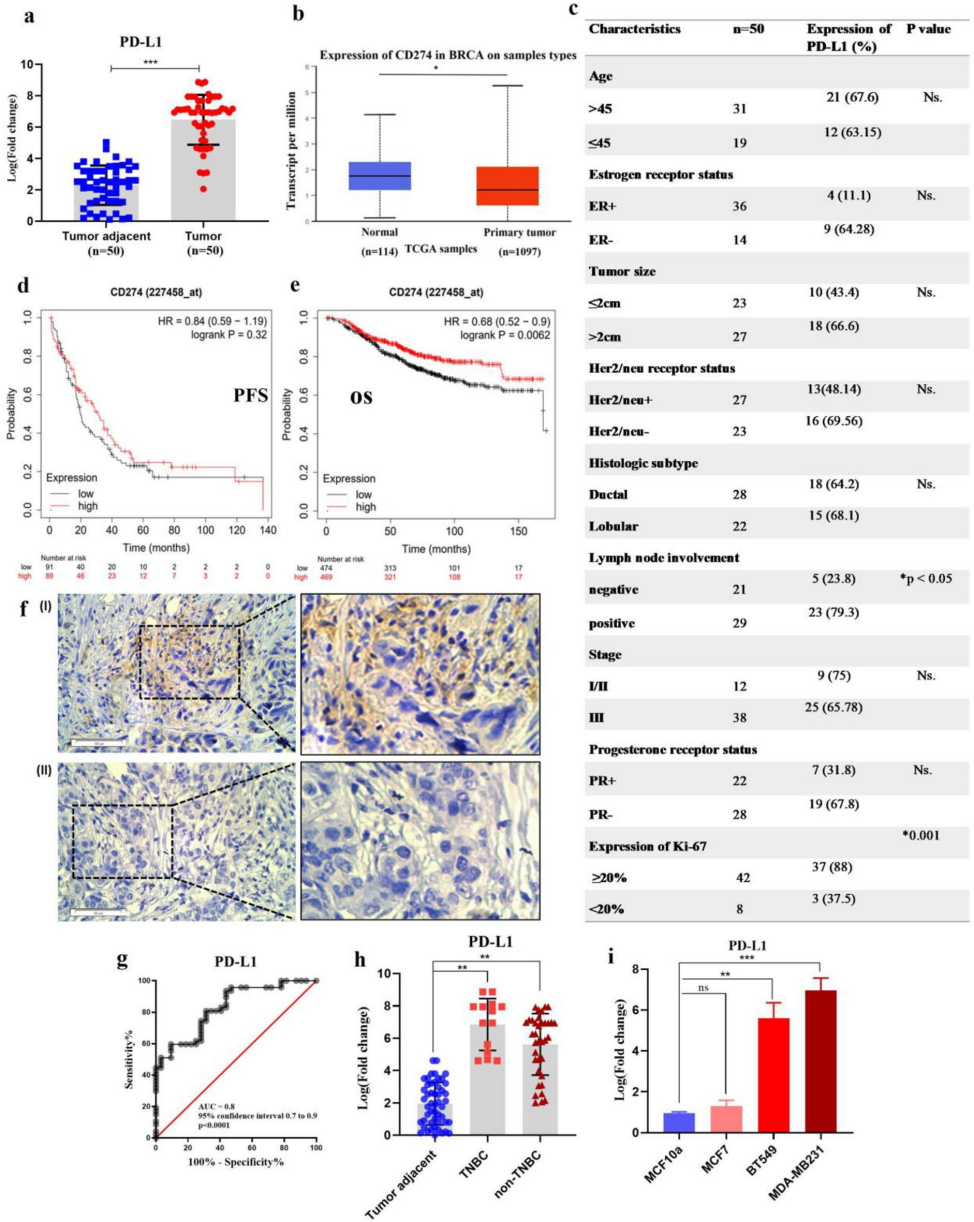
PD‐L1 expressions were analyzed in BC tissue samples and cell lines. (**a**) PD-L1 expression was upregulated in BC tissues (n = 50) as compared with normal samples using qRT‐PCR (**b**) Shows PD-L1 mRNA expression in normal and breast cancer tissues using TCGA database (**c**) The relationship between PD-L1 expression and clinical pathologic features (**d**) The Kaplan–Meier plotter shows the prognostic role of PD-L1 mRNA expression in progression-free survival (**e**) The Kaplan–Meier plotter for overall survival shows the prognostic role of PD-L1 mRNA expression in breast cancer (**f**) Immunohistochemical staining of PD-L1 (I) in breast cancer tissue and (II) in breast non-cancerous tissue (**g**) Area under the curve (AUC) and receiver operating characteristics (ROC) for PD-L1 (**h**) The correlation between PD-L1 expression with TNBC and non-TNBC status of the tumor (**i**) PDL1 expression in BC cell lines compared to normal breast cell line; ***P ≤ 0.001; **P ≤ 0.01; ns = non-significant. The results were expressed as the mean ± SE of the experiments. PD‐L1, programmed death-ligand 1; qRT‐PCR, quantitative reverse transcription-PCR; TP, Tumor Patient; NTP, Non-Tumor Patient.

First, we analyzed PD-L1 mRNA expression in 50 breast cancer and noncancerous tissues. PD-L1 was upregulated (4.297 ± 0.4107) in breast cancer compared to noncancerous tissue (Fig. 1a). PD-L1 expression was evaluated based on the TCGA database to assess its prognostic significance in breast cancer. The database showed breast cancer tumors have higher expression than normal tissue. (P = 4.930500E−02) (Fig. 1b). PD-L1 expression was unrelated to patient age, estrogen receptor status, tumor size, etc. (P > 0.05). PD-L1 positive expression was higher in patients with lymph node metastases, and Ki-67 expression was 20% (P < 0.05) (Fig. 1c). Next, PD-L1 mRNA in human breast cancer was assessed using the Kaplan–Meier Plotter online database. An online KM plotter database using microarray data showed that PD-L1 mRNA expression was not significantly correlated with PFS (Progression-Free Survival) in 458 breast cancer patients (HR = 0.84, 95% CI = 0.59–1.19, P = 0.32) (Fig. 1d). In 1880 breast cancer patients, PD-L1 mRNA was associated with overall survival (HR = 0.68, 95% CI = 0.52–0.9, P = 0.0062) (Fig. 1e). We determined the expression of PD-L1 protein in BC tissues by immunohistochemistry. The presence of PD-L1 protein was high in breast cancer samples (Fig. 1f). In this study, we used ROC curves (AUC = 0.8, 96% sensitivity, and 44% specificity) to establish the predictive significance of these changed expression levels separately (Fig. 1g). According to Fig. 1h, we showed that there is a significant relationship between PD-L1 expression and TNBC or non-TNBC status of patients participating in this study. As shown in Fig. 1i, MDA-MB231 and BT549 have higher PD-L1 levels than normal breast tissue cells (MCF10a). PD-L1 expression may be diagnostic for BC based on these data.

### MiR-335 and miR-145 can target the 3′-UTR of PD-L1

**Figure 2:**
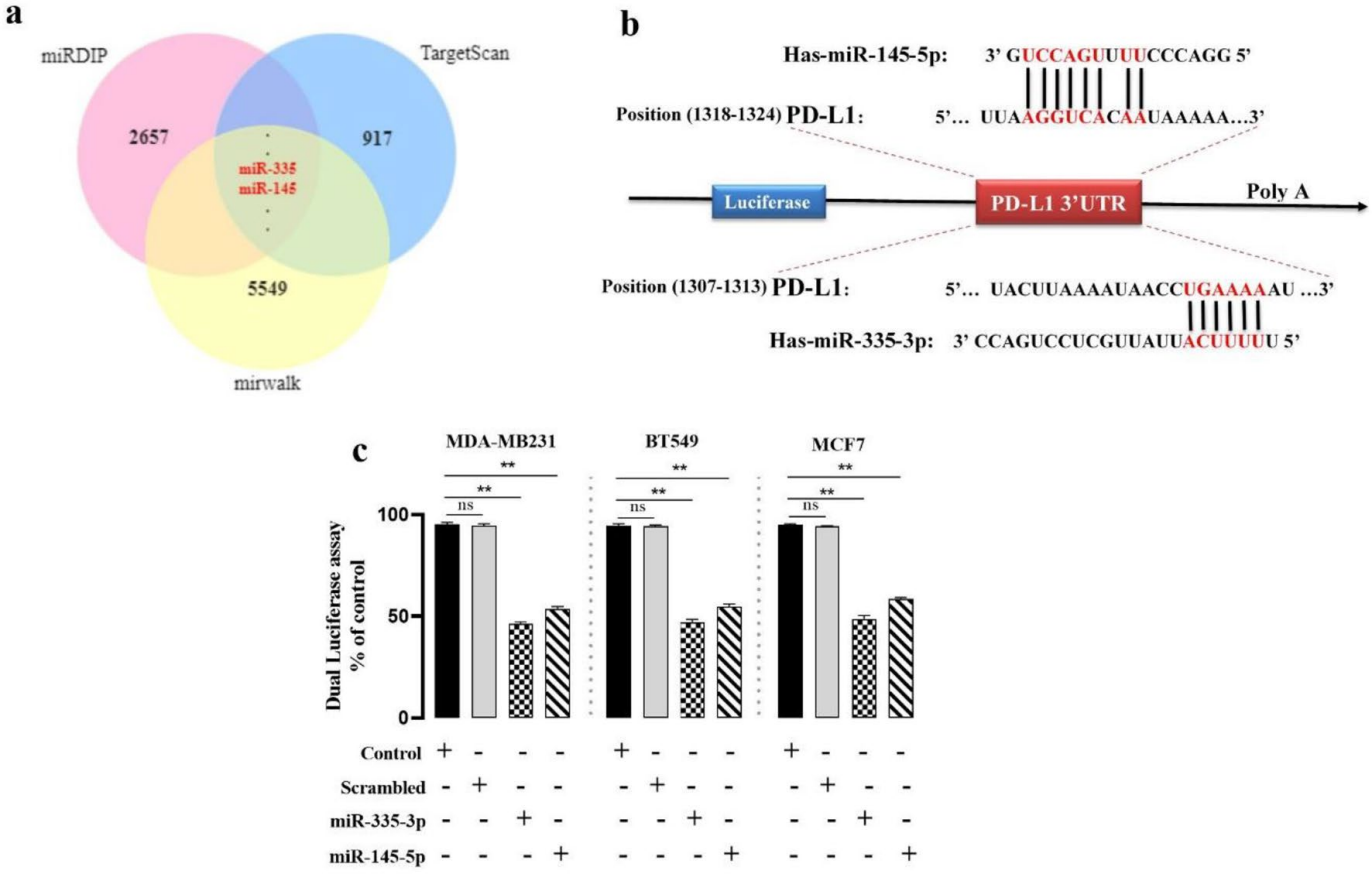
PD-L1 is a direct target of miR-335 and -145 in breast cancer cell lines. (a) MiR-335 and -145 downstream target genes predicted by miRDIP, target scan, and miRwalk (**b**) miR-335 and -145 are highly conserved across species and have binding sites within the 3′-UTR of human PD-L1 (**c**) MDA-MB231; BT549; and MCF7 cells were transfected with renilla and firefly luciferase expression constructs from psiCHEC KTM-2 harboring PD-L1 3′-UTR and miR-335 and -145, or scramble miR. Dual-Luciferase assays were done after 48 h. The relative Renilla luciferase activity went down, but the scramble miR had no effect. ns = non-significant, **P ≤ 0.01, ***P ≤ 0.001.

We searched for miR binding sites in the 3′-UTR of PD-L1 using miRDIP (http://www.ophid.utoronto.ca/), miRwalk (http://www.mirwalk.umm.uni-heidelberg.de/), and TargetScan 4.0 (http://www.targetscan.org/). The 3′-UTR of the PD-L1 gene was complemented by miR-335 and miR-145 (Fig. 2a, b). To confirm these miR-target contacts, we cloned the PD-L1 complementary site into psiCHECKTM-2. This plasmid was co-transfected into MDA-MB231, BT549, and MCF7 cells. In Fig. 2c, miR-335 and miR-145 reduce luciferase activity relative to scramble. Transfecting miR-335 into MDA-MB231, BT549, and MCF7 cell lines decreased luciferase activity to 40% ± 0.571%, 41.67% ± 0.68%, and 36.34% ± 0.11% (P ≤ 0.05). Transfecting miR-145 into MDA-MB231, BT549, and MCF7 cell lines decreased luciferase activity to 47.66% ± 0.64%, 49% ± 0.2% and 46.33% ± 1.29% correspondingly (P ≤ 0.05).

### Prognostic values of miR-335 and -145 in breast cancer and in silico exploration of their target genes

**Figure 3:**
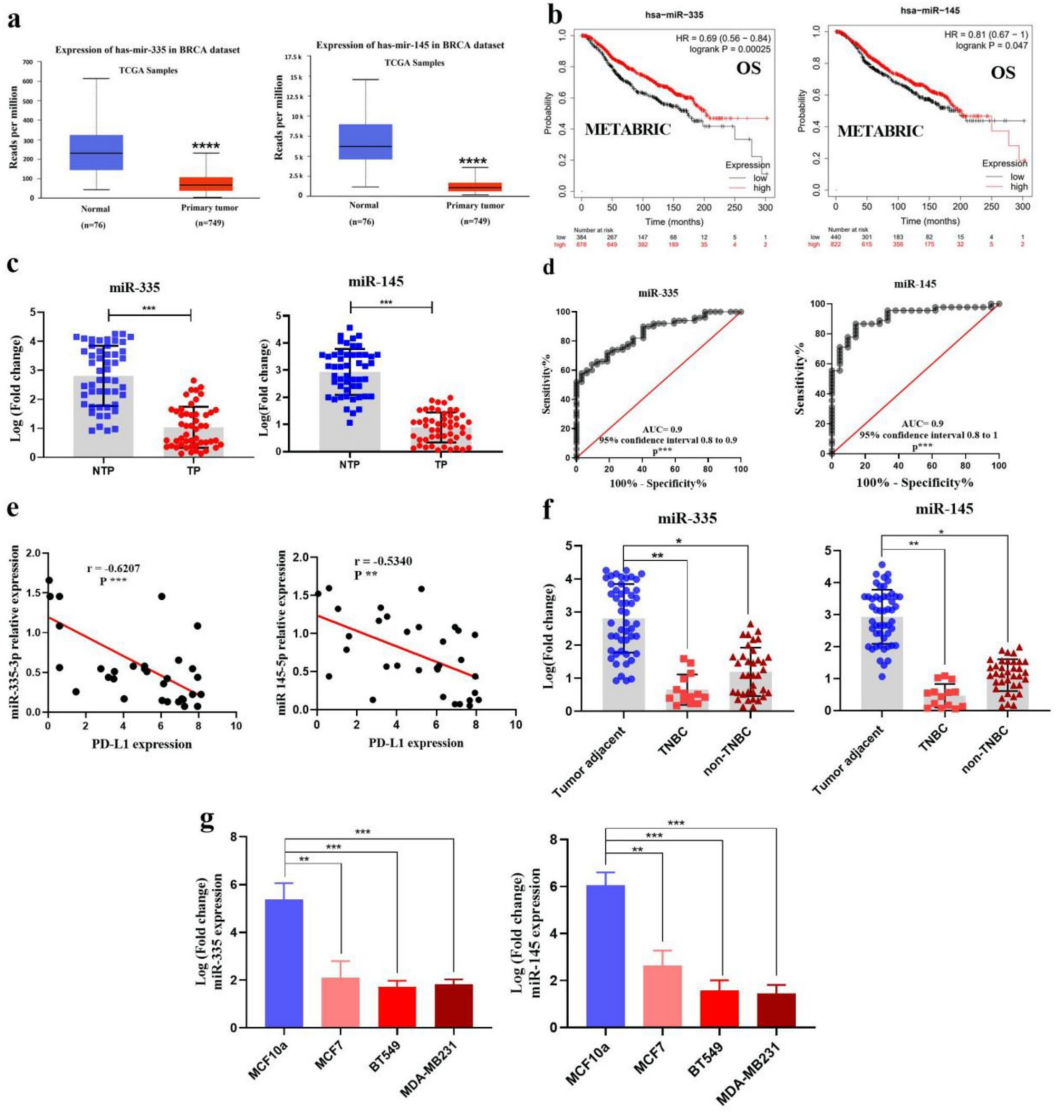
miR‐335 and -145 expressions were analyzed in BC tissue samples and cell lines. (**a**) Analysis of miR-335 and -145 expression in BC via TGCA samples (**b**)  The Kaplan–Meier plotter shows lower levels of miR-335 and -145 expression in primary breast tumors are associated with stronger and poorer, respectively, overall survival in the METABRIC (n = 1078) breast cancer patient datasets (**c**) miR‐335 and -145 expression were downregulated in BC tissues as compared with normal samples using qRT‐PCR (**d**) Receiver operating characteristics (ROC) and area under the curve (AUC) for miR-335 and -145 (**e**) Decreased expression of miR-335 and-145 was correlated with PD‐L1 mRNA level (r = − 0.6207, r = − 0.5431, respectively, Pearson correlation) (**f**) The correlation between miR-335 and-145 expression and the TNBC or non-TNBC status of the tumor (**g**) Expression of miR-335 and -145 in BC cell lines compared to normal breast tissue samples; ***P ≤ 0.001; **P ≤ 0.01; ns = non-significant. The results were expressed as the mean ± SE of the experiments. BC, Breast Cancer; mRNA, messenger RNA; PD‐L1, programmed death-ligand 1; qRT‐PCR, quantitative reverse transcription-PCR.

To evaluate the prognostic values of miR-335 and -145 in breast cancers, the expression of these miRs was assessed based on the breast cancer TCGA miRNA database. In the database, significantly lower expression was observed in breast cancer tumors (n = 749) compared to normal tissue (n = 76) (P = 2.098e−09, < 1e−12, respectively) (Fig. 3a). Furthermore, a breast-cancer survival analysis was performed based on the breast-cancer METABRIC miRNA database. The results showed that the low expression of miR-335 and -145 was significantly correlated with overall survival rate (BC, P = 0.00025 and P = 0.047, respectively), which demonstrates its biological importance (Fig. 3b). Based on this analysis, miR-335 and -145 and their target genes might potentially be considered biomarkers for breast cancer prognosis. In this study, miR-145 and 335 were significantly downregulated (0.736 ± 0.087, 0.606 ± 0.09 respectively) in breast cancer tissue compared with noncancerous tissue (Fig. 3c). We also determined the predictive value of these altered miR-335 and -145 expression levels using ROC curves (AUC = 0.9) (Fig. 3d). As compared with noncancerous patients with low PD-L1 levels, BC cases with high PD-L1 levels showed low miR-145 and -335 levels, with Pearson correlation analyses revealing a clear inverse relation between miR-145 and -335 expression and PD-L1 mRNA level (r =  − 0.5431; P-value = 0.0013, r =  − 0.6207; P-value = 0.0002 respectively) (Fig. 3e). We showed a significant correlation between miR-335 and -145 expression and the TNBC or non-TNBC status of study participants (Fig. 3f). As shown in Fig. 3g, miR-335 and-145 were more downregulated in breast cancer cell lines, with the highest expression level of PD-L1 compared with the normal breast tissue cell line (MCF10a).

### Hypermethylation of miR-335 and miR-145 promoters might be responsible for upregulation of PD-L1

**Figure 4:**
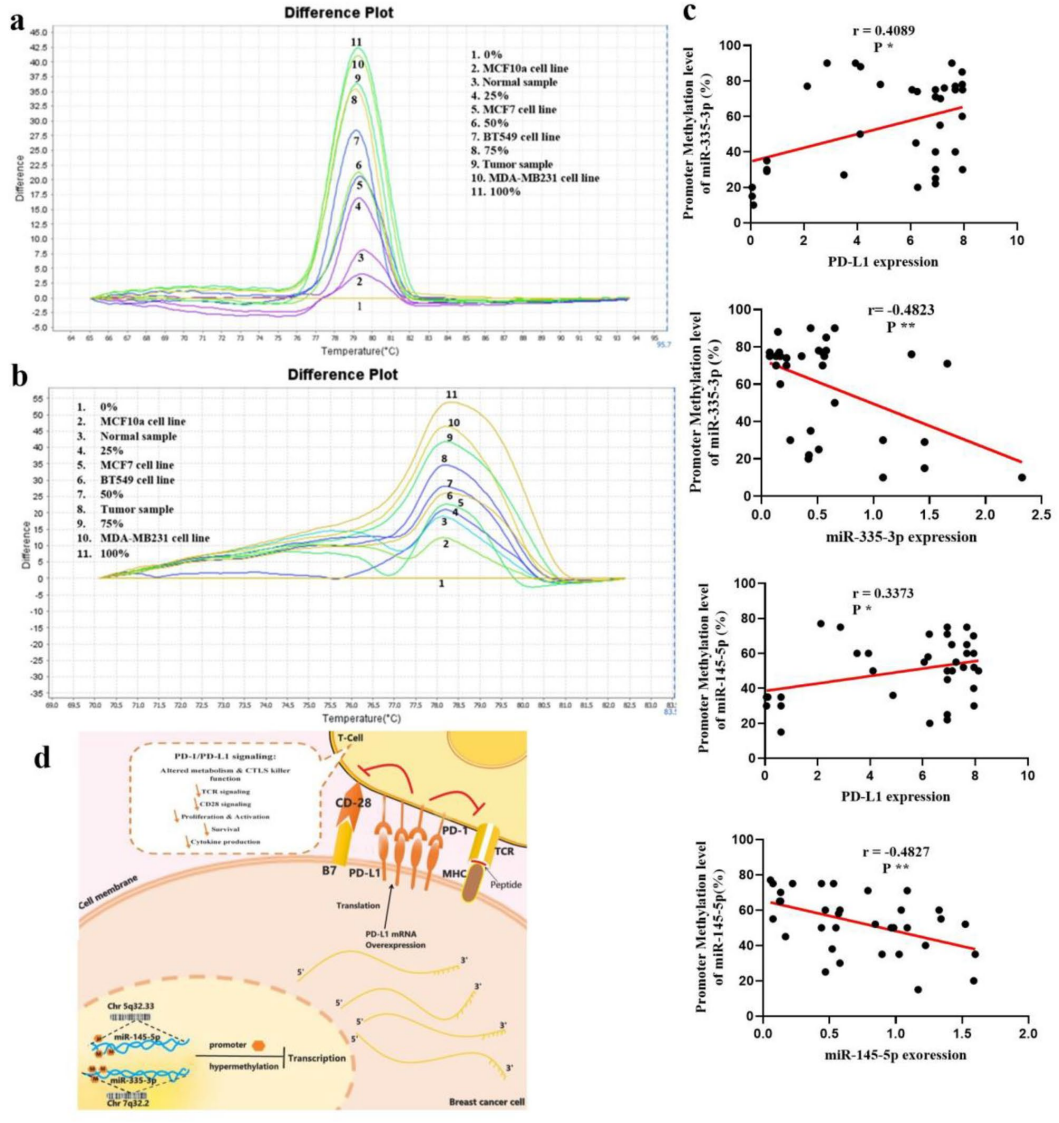
High-resolution melting of miR-335 and -145 genes. (**a**) Templates with different ratios (from 100 to 0%), some BC tissue samples, and methylated DNA cell lines were amplified by miR-335 and (**b**) miR-145 specific primers, and HRM analysis was done (**c**) Correlation analysis between the relative PD-L1 expression level and average methylation level of miR-335 and -145 promoters (r = 0.4089 and r = 0.3373, respectively, Pearson correlation). Relative miR-335 and-145 expression levels and the average methylation level of miR-335 and-145 promoters (r = − 0.4823 and r = − 0.4827, respectively, Pearson correlation) (**d**) A schematic showing how hyper-methylation of the miR-335 and -145 promoters can turn off T cells. In breast cancer cells, PD-L1 expression increases due to promoter hyper-methylation of PD-L1 tumor suppressor miRs, such as miR-335 and -145, and their lack of transcription. PD-L1, by binding PD-1, blocks the activation of T cells. *P ≤ 0.05, **P ≤ 0.01, ***P ≤ 0.001.

Another question is why down-regulation of miR-335 and -145 occurs in the malignant transition of breast cancer cells. Tumor suppressor miRs, including miR-335 and -145, have been reported to be extinguished by abnormal DNA hypermethylation. Analysis of the miR-335 and -145 promoter showed three CpG islands upstream of the transcriptional start site, indicating that it may regulate the mature miR-335 and -145 and its precursor through DNA methylation. BC tissues showed hypermethylation in the miR-335 and -145 promoters, whereas the methylation levels of miR-335 and -145 were lower in noncancerous tissues (Table 1). We also determined the methylation rate in the miR-335 and -145 promoters in BC cells. MDA-MB231 cells had the highest methylation level, and MCF7 cells had the lowest level of methylation in the miR-335 and -145 promoters (Fig. 4a, b). Statistical analysis also showed that the level of methylation was strongly linked to the expression of miR-335, miR-145, and PD-L1 in both cancerous and healthy tissues (Fig. 4c). The surface of an activated T cell contains PD-1. Both immunological and cancerous cells express the protein PD-L1. By attaching to PD-1, PD-L1 prevents T-cell activation (Fig. 4d).

**Table 1:** Methylation level of tumor suppressor miR-335 and -145 promoter in cell lines, breast cancer samples and normal tissues.

Sample	miR-335 (%)	miR-145 (%)
TP	(n = 15) 75%	(n = 9) 75%
TP	(n = 30) 75%-100%	(n = 6) > 75%
TP	(n = 5) 50%-75%	(n = 35) < 75%
NTP	< 25%	< 25%
MDA-MB231	75%-100%	> 75%
BT549	25%-50%	< 50%
MCF7	25%-50%	> 25%
MCF10a	< 25%	< 25%

TP, tumor patient; NTP, non tumor patient.

### Down-regulation of PD-L1 mRNA in BC cell lines following miR-335 and -145 transfection

**Figure 5:**
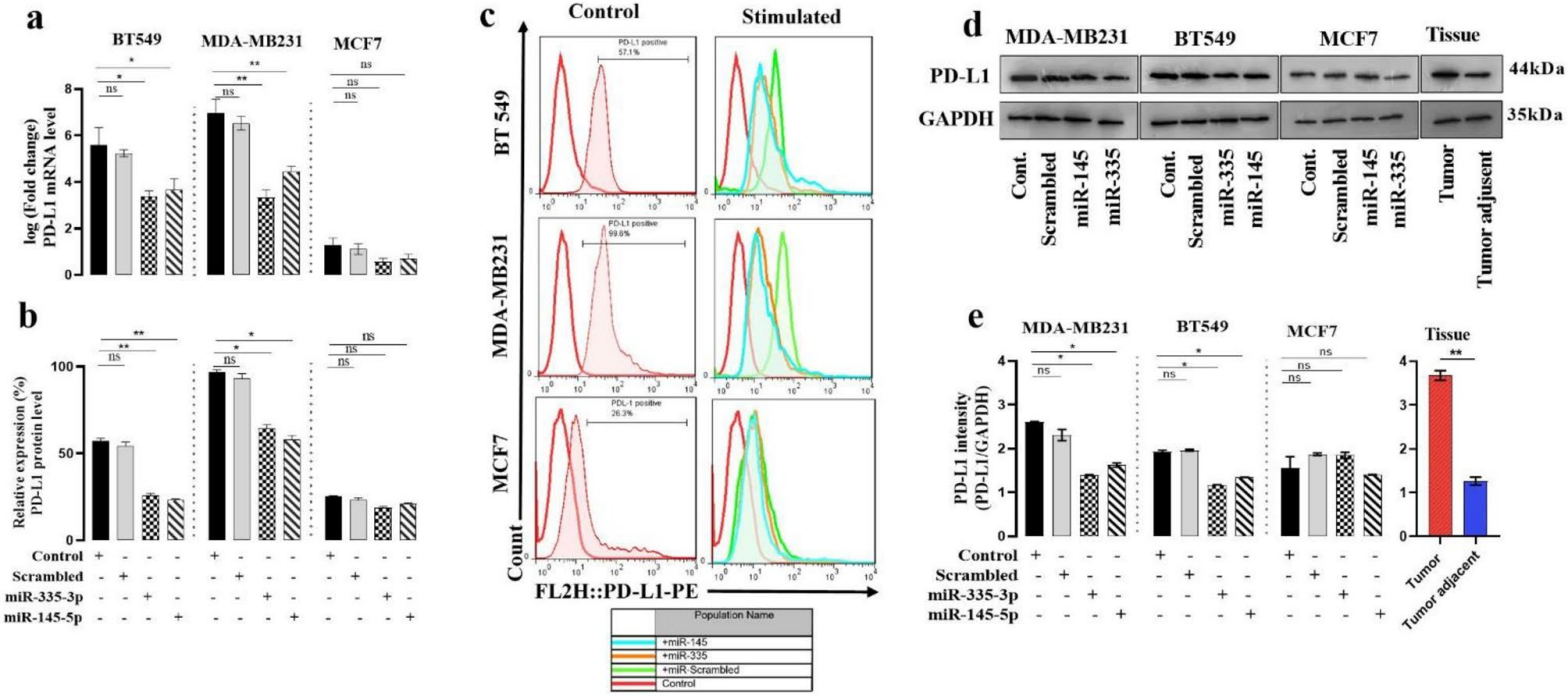
The effect of overexpression of miR-335 and -145 in the control of PD-L1 expression in mRNA and protein levels. (**a**) PD-L1 expression levels through miR‐335 and -145 transfection were analyzed using qRT‐PCR. The results showed that miR‐335 and -145 exogenous overexpression could significantly reduce PD-L1 expression at the mRNA level in BC cell lines. (**b**) Graphs represent PD‐L1 protein expression levels. (**c**) FACS analysis of PD‑L1 in breast cancer cell lines. Anti‑human PD‑L1 was used as a binding antibody to cell‑surface PD‑L1 protein. (**d**) Western blots showing changes in expression levels of PD-L1 protein after miRNA transfection in breast cancer cell lines and tissue. (**e**) Statistical histogram of the western blots (n = 3). GAPDH was used as a loading control. All data were presented as mean ± SEM. *P ≤ 0.05, **P ≤ 0.01, ***P ≤ 0.001.

In the next step, to determine whether miR-335 and -145 regulate the expression level of the PD-L1 gene by altering the promoter methylation status of these miRs, we examined the expression levels of these genes. To confirm the hypothesis that overexpression of miR-335 and miR-145 down-regulates PD-L1 mRNA expression in BC cell lines, miR-335, -145, or scramble oligonucleotides were transfected into MDA-MB231, BT549, and MCF7 cell lines. Afterward, the levels of PD-L1 mRNA were measured by quantitative real-time PCR. The overexpression of miR-335 and -145 significantly reduced the level of PD-L1 mRNA in MDA-MB231 and BT549 compared with non-transfected cells (Fig. 5a). In MCF7 cells, no significant change was seen in the expression level of the PD-L1 gene.

### MiR-335 and miR-145 reduce PD-L1 protein expression in the BC cell

We looked at how surface PD-L1 expression differed between breast cancer cell lines after transfection with miR-335 and -145. Compared to the control group, flow-cytometry results revealed that miR-335 and -145 transfection could significantly decrease the PD-L1 protein expression of BC cell lines. The overexpression of miR-335 and -145 after 48 h reduced the level of PD-L1 protein in MDA-MB231 to 38.3%  ± 1.2% and 41.5% ± 1.8% respectively, BT549 to 37.7% ± 2.5% and 35% ± 1% respectively, and in MCF7 to 7.7% ± 0.5% and 4.5%  ±  0.2%, respectively, compared with non-transfected cells (Fig. 5b, c). Western blot results confirmed that PD-L1 proteins were decreased by targeted miRNAs 335 and 145 in MDA-MB231 and BT549 cells. Meanwhile,  PD-L1 protein expression levels in breast cancer tissue were significantly increased (Fig. 5d, e).

### MiR-335 and miR-145 induce apoptosis in cell lines

**Figure 6:**
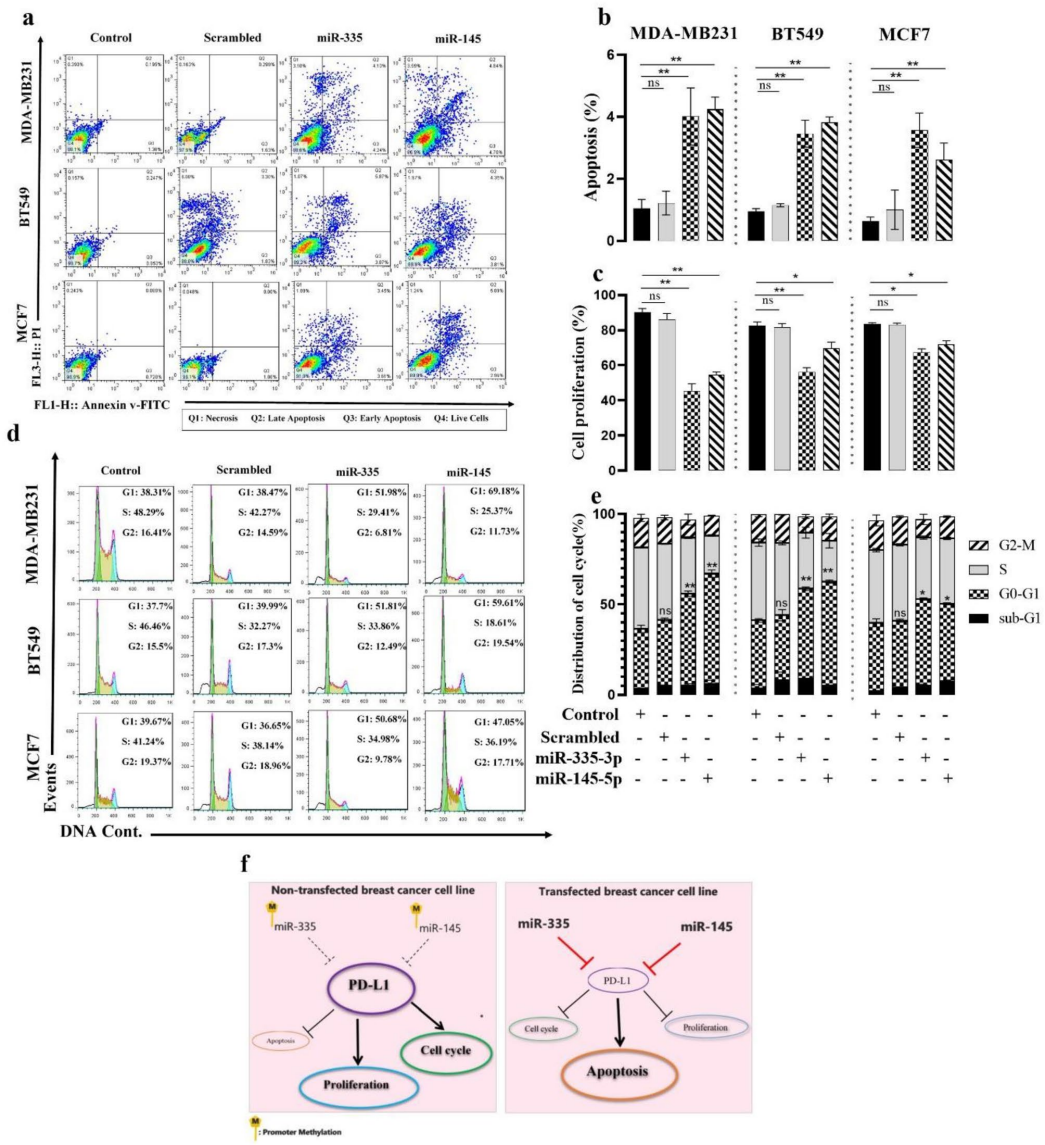
MiR‐335 and -145 effects on BC cell apoptosis, proliferation, and cell cycle. (**a**) Apoptosis induction was investigated using flow cytometry. (**b**) Apoptosis rates are shown in the graphs; miR-335 and -145 transfection significantly increased apoptosis in MDA-MB231, BT549, and MCF7 cells. (**c**) A statistically significant reduction in MDA-MB231, BT549, and MCF7 cell proliferation was observed in the miR-335 and -145 groups when compared to the control groups. No difference was found between the control group and the scrambled group. (**d**) Analysis of the cell cycle distribution of PD-L1 expression due to overexpression of miR-335 and-145 could arrest the cell cycle at the G0/G1 phase. (**e**) The graphs show the percentage of cells in each cell cycle phase. (**f**) Schematic graph depicting the function of miR-335 and -145 in breast cancer. MiR-335 and -145 overexpression leads to PD-L1 inhibition, which leads to enhanced proliferation and the cell cycle while inhibiting apoptosis in breast cancer. Overexpression of miR-335 and -145 reinforces the suppression of its target PD-L1, leading to the suppression of tumorigenicity in breast cancer. *P ≤ 0.05, **P ≤ 0.01, ns = non-significant.

To further illustrate PD-L1 as a direct target of miR-335 and -145, we examine the significance of PD-L1 in miR-335 and -145-mediated cell survival. Using the Annexin V/PI assay, we also investigated the effect of these miRs on apoptosis induction. As shown in Fig. 6a, b, miR335 and -145 could increase the apoptosis rate in MDA-MB231 to 2.96% ± 0.38% and 3.19% ± 0.06%, respectively; in BT549 to 2.5% ± 0.2% and 2.62% ± 0.1%, respectively; in and MCF7 cell lines to 2.93% ± 0.4% and 1.9% ± 0.1%, respectively, when compared to the control groups. These results further suggest PD-L1 as a major target of miR-335 and -145.

### Overexpression of miR-335 and -145 inhibits BC cell lines proliferation and cell cycle

To understand the functional role of miR-335 and -145 on BC cell lines, we evaluated the impact of these miRs on the cellular proliferation of MDA-MB231, BT549, and MCF7 cell lines. As shown in Fig. 6c, 48 h after transfecting miR-335 and -145, the proliferation of these cells in comparison with non-transfected cells was decreased, in MDA-MB231 to 52.3% ± 2.4% and 33% ± 1.1%, respectively; in BT549 to 35.3% ± 0.9% and 23% ± 0.4%, respectively; and in MCF7 to 10.3% ± 0.3% and 14.4% ± 1.7% (mean ± SE), respectively, which was significantly different from non-transfected cells and transfected cells with scrambled miR. The cell cycle found by FCM also showed that overexpression of miR-335 and -145 stopped cells in the G0/G1 phase to stop the growth of cancer cells (Fig. 6d, e). In this study, we investigate miR-335 and -145 promoter hypermethylation in breast cancer. MiR-335 and -145 promoter hypermethylation is higher in breast cancer cell lines and malignant tissues. PD-L1 suppresses apoptosis and promotes breast cancer cell proliferation and the cycle. MiR-335 and -145 regulate PD-L1, which contributes to pro-tumor effects (Fig. 6f).

## Discussion

Breast carcinogenesis is a process involving the dysregulation of tumor suppressor genes and oncogenes^33^. The PD‐1/PD‐L1 pathway is important as an immunosuppressant, and it is deregulated in a wide range of human cancers^34^, including BC. PD-L1 is involved in tumorigenesis and immunosuppression, making it a valuable therapeutic target for a variety of human cancers^35–37^. The correlation between decreased T-cell proliferation and increased apoptosis and tumor immune escape, with increased expression of PD-L1 proteins on cancer cells, creates an understanding of controlling PD-L1 expression using cancer cells as a model. In addition, significant advances in cancer therapy have been made in early clinical trials using Food and Drug Administration (FDA)-approved antibodies that target PD‐1/PDL1, such as pembrolizumab^38^. However, to increase the number of patients benefiting from the blockade of the safety checkpoint, these inhibitors are more commonly prescribed in combination with other therapies, including chemotherapy. Nevertheless, cumulative studies have shown that many common chemotherapy agents, including 5‐Fluorouracil and Paclitaxel, lead to rearrangement of PD‐L1. This phenomenon, in turn, reduces cell‐mediated antitumor T reactions and promotes immune system escape^39^. Therefore, identifying the molecular mechanisms involved in regulating the PD‐1/PD‐L1 pathway through breast tumorigenesis could help improve new strategies aimed at the antitumor immune response against BC. This study's analysis of clinical samples showed that PD-L1 expression was higher in BC tissue than in control samples. In the current study, we investigated the expression level of PD-L1 and its relationship with survival parameters using publicly available data and a variety of bioinformatics methodologies. Based on what we found, PD-L1 expression levels were strongly linked to shorter patient survival times and were much higher in BC tumor tissue than in normal tissue. High expression of PD-L1 was also significantly associated with lymph node metastases, and Ki-67 expression was 20%. Among the 50 cases of BC patients included in this study, immunohistochemically stained results showed that PD-L1 was positive in BC patients, which was brownish.

In the context of epigenetic regulation of the genome generally, promotor methylation is a distinct and reversible mechanism for gene silencing. Accordingly, promoter hypermethylation of tumor suppressor genes is found in many human cancers. MiRNAs are another epigenetic mechanism for controlling definitive gene expression because the translation of transcripts is expressly abrogated via mRNA degradation after miRNA binding. In this study, we found that BC is associated with low expression of miR-335 and -145. MiR-335-3p was found to be significantly downregulated as a tumor suppressor in cancers such as gastric cancer^40^, multiple myeloma^41^, human glioma^42^, etc. Furthermore, miR-145-5p downregulation has been found in a series of human cancers. Previous studies have highlighted its role as a tumor suppressor by modulating multiple oncogenes in cancer cells^43^, including breast cancer cells^44^. MiR-335 and -145 expression differs in benign and malignant tumors in women^44,45^. Furthermore, miR-335 and -145 were found to suppress the oncoproteins PD-L1, inhibiting the growth of breast cancer cells^46,47^. In the present study, using bioinformatics analysis, we identified the 3′‐UTR PD‐L1 region as a potential target for miR‐335 and -145 targeting, suggesting its possible role in the escape of immune tumor cells. Subsequently, the analysis of clinical samples showed that the expression of miR-335 and -145 in tumor tissues was significantly lower than that in adjacent tissues, and its expression level was negatively correlated with PD-L1, suggesting that miR‐335 and -145 may be involved in post-transcriptional regulation of PD‐L1 expression. With this in mind, we used the luciferase assay, which confirmed that miR‐335 and -145 could target specific regions of the PD‐L1 3′‐UTR and significantly coordinate its expression. In BC, several miRs undergo transcriptional inactivation by hypermethylation of their promoters. This can lead to the overactivity of their oncogenic targets^48^. CpG island hypermethylation-mediated silencing of miR-34b/c, miR-148, and miR-9-3 is correlated with the loss of oncogenic target gene regulation, such as C-MYC, E2F3, CDK6, and TGIF2, and promotes invasion and metastasis^49^. We detected the promoter hyper methylation status of miR-335 and -145 in BC tissues and cell lines through HRM. We discovered that BC tissues and cell lines, particularly metastatic cell lines, had abnormally high levels of methylation compared to normal tissues and cells; miRNA methylation levels were positively associated with PD-L1 expression in BC patients. We analyzed the correlation between miR-335 and -145 methylation status and PD-L1 expression in BC tissues. We showed that up-regulated expression of PD-L1 was significantly correlated with CpG island DNA hyper methylation in the promoter region of miR-335 and -145. In addition, DNA is less prone to degradation than RNA, frequency, stability, and variability among patients, which may indicate clinical usefulness. So, we think that, in addition to miRNA expression, abnormal methylation of the miR-335 and -145 promoters is another great epigenetic tumor marker. Unlike genetic modifications, the reversibility of epigenetic changes makes them a potential therapeutic target. The previous study results indicated that the expression of some silenced miRs in cancer cells could be restored by treatment with demethylating agents like 5-aza-2′-deoxycytidine, which inhibits the growth, invasion, and metastasis of cancer cells^50^. The US Food and Drug Administration (FDA) has approved 5-azacytidine (Azacitidine)^46^ and 5-aza-2-deoxycytidine (Decitabine)^51^, which modify DNA methylation, for the treatment of patients with MDS.

Previous reports of miR-335 and -145's involvement in human cancer have confirmed their role as a regulator of cancer cell growth and invasion^52,53^ and the correlation between metastasis and human cancer. Exogenous overexpression of miR-335 and -145 in BC cells led to a big drop in PD-L1 expression at both the mRNA and protein levels. This showed that miR-335 and -145 change how the PD-L1 gene is expressed in BC.

This study has highlighted the efficacy of miR-335 and -145 in restraining tumor proliferation, arresting the cell cycle, and stimulating tumor apoptosis in BC cells. Cancer proliferation and progression are mainly associated with the ability of anti-tumor immunity to evade cancer cells^54^. Ghebeh et al.^55^ have shown that the expression of PD-L1 in tumors could be linked to the development of breast cancer.

According to recent studies, PD-L1 plays a specific tumor-intrinsic role in promoting cancer metastasis, development, and treatment resistance^56^.Our study demonstrated that downregulated PD-L1 expression, through overexpression of candidate miRs, in BC cell lines significantly inhibited cell proliferation along with induction of G0/G1 phase arrest and apoptosis induction. The findings in other human tumors were in accordance with these results. In human AML cancer, the PD-L1 expression level was linked to a high number of cancer cells that were dividing^57^, and it has been shown that too much PD-L1 expression makes tumor cells grow^58^. Additionally, suppressing the expression of PD-L1 in gastric cancer cells could significantly increase apoptosis and reduce invasion, migration, and cell proliferation^59^. Moreover, in the present study, miR‐335 and -145 were shown to induce apoptosis in BC cells by altering their significant regulators' expression.

These findings suggest that the methylation status and expression levels of miR-335 and -145, and PD-L1 overexpression as a consequence, may serve as markers of BC tumors. Further studies on the epigenetic regulation of miRNA expression are necessary, and the regulation of miRNA expression by epigenetic drugs might be of great promise for BC prevention and therapy.

## Materials and methods

### Clinical tissue samples and cell lines

50 breast cancer patient samples were obtained from Khatam-al-Anbya Hospital in Tehran, Iran. The hospital records included sex, age, lymph node metastases, and clinical grade (Fig. 1c). Radiotherapy or chemotherapy patients before surgery were excluded. A pathologist without patient data diagnosed tumor samples using the WHO’s approach. The Pasteur Institute of Iran authorized this work (Ethical code: IR.PII.REC.1398.023). Subjects gave informed consent. Patient data was analyzed for demographic and clinical information. Three human BC cell lines (MCF-7, BT-549, and MDA-MB-231), and a breast normal cell line (MCF-10a), were used in this investigation. MDA-MB-231 is extremely aggressive, invasive, and poorly differentiated and lacks estrogen receptor (ER), progesterone receptor (PR), and HER2 (human epidermal growth factor receptor 2) expression. BT-549 cells are epithelial cells isolated from a papillary, invasive ductal tumor and are characterized as triple-negative/basal-B breast mammary carcinoma. MCF-7 has estrogen, progesterone, and glucocorticoid receptors. MCF-7 is an adherent epithelial luminal cell line positive for estrogen and progesterone receptors. They are hormone-dependent, yet serum steroid hormones are enough to develop cells. MCF10A is the most often used normal breast cell model. These breast tissue cells spontaneously immortalized. They are non-tumorigenic and estrogen receptor-negative. All cell lines were grown in a 37 °C humidified incubator with 5% CO2 and DMEM with 10% fetal bovine serum and 1% penicillin/streptomycin (Sigma Aldrich, St. Louis, MO, USA).

### Kaplan–Meier overall survival analysis

To assess the prognostic value of the PD-L1 gene, miR-335, and miR-145 in breast cancer patients, the Kaplan–Meier Plotter website (Budapest, Hungary) (http://kmplot.com/analysis/) (accessed May 14, 2022) was utilized^60^. For this purpose, PD-L1, miR-335, and 145 Kaplan–Meier plots for breast cancer patients were generated. Log rank p-values of 0.05 were considered statistically significant for the Kaplan–Meier (KM) plots of all genes.

### Immunohistochemistry using the PD-L1 antibody

At least three serial sections of formalin-fixed, paraffin-embedded BC tissues were placed on positively charged glass microscope slides. For IHC, Khatam-al-anbia Pathology Lab used a technique refined and validated for research specimen testing with PD-L1 monoclonal antibody (Master Diagnostica, Spain). For IHC, Khatam-al-anbia Pathology Lab used a technique optimized and validated for research specimen testing with PD-L1 monoclonal antibody (Master Diagnostica, Spain). Similarly, 100 μl of rabbit monoclonal anti-PD-L1 antibody at 200 × dilution was incubated for 60 min at room temperature, washed, subsequently incubated with HRP- and anti-rabbit antibody-conjugated polymer for 8 min, washed, incubated with hydrogen peroxide reagent, washed, and finally incubated with DAB substrate for signal development.

### Dual Luciferase assay

MiR-335, miR-145, and target gene 3′-UTR interaction was demonstrated using a dual-luciferase assay. The 3′-UTR of PD-L1 and a special scramble sequence (AAGCTTCATAGGGCATAGC) as a negative control were cloned into the psiCHECKTM-2 vector (Promega, C8021) to perform luciferase reporter assays (PCR primers are listed in Table 2). BC cells were transfected with a psiCHECK-2 plasmid containing PD-L1 binding site mutations at positions 1307-1313 and 1318-1324, as well as a negative control or a miR-335 and-145 mimic. In 48-well plates, BT549, MDA-MB231, and MCF7 cells were co-transfected with psiCHECK-2 vectors containing the 3′-UTR of PD-L1 or a scramble sequence and 100 nM of each precursor miR (335, 145, or scramble miRs) using HyperFect (Qiagen, Germany). Renilla luciferase activity was adjusted to firefly luciferase. At 24 h, 48 h, and 72 h after transfection, luciferase activity was assessed (Promega, USA).

**Table 2:** Relative primers used for PCR, real-time PCR, stem-loop sequences and HRM.

Primer Sequences
**Gene Sequence of oligonucelotides from 5′ to 3′ for PCR analysis (3’UTR)**
*PD-L1* Forward: CCGCTCGAGCAGATACACATTTGGAGGAGA Reverse: ATAAGAATGCGGCCGCTGTGCAGGATTCAAAAGCAT
**Gene Sequence of oligonucelotides from 5′ to 3′ for real time-PCR analysis**
*PD-L1* Forward: GTGATCCGCTGCATGATCAG Reverse: GGTAGCCCTCAGCCTGACATG
*miR-335* Forward: CCAGTTTCATTATTGCTCCTGACC Reverse: AGACTGCACCTGTCCGG
*miR-145* Forward: GTCCAGTTTTCCCAGGAATCCC Reverse: AGACTGCACCTGTCCGG
Stem loop *miR-335* CAATTAGACTACACCTGTCCGGTCCCTGCGTCCTGTAGTCTAATTGGGTCAG
Stem loop *miR-145* CAATTAGACTACACCTGTCCGGTCCCTGCGTCCTGTAGTCTAATTGAGGGAT
**Gene Sequence of oligonucelotides from 5′ to 3′ for HRM analysis**
*miR-335* Forward: TGGTGGGTTTAATAGAGTTTGTTG Reverse: CACAAACCCCACAAAAAAATAAC
*miR-145* Forward: TGGTAGGAGATTGGGGAATATATAT : Reverse: TTCTACATCCAACCCCATCTATAAC

### RNA isolation, cDNA synthesis, and quantitative real-time PCR

RiboPureTM kit extracted total RNA (Life Technology, MA, and USA). The RNA concentration was quantified by a spectrophotometer (IMPLEN, Munich, Germany), and quality was determined by the 260/A280 absorbance ratio. Using 1 g of RNA as a template and a cDNA synthesis kit, the M-MuLV reverse transcriptase enzyme generated cDNA (Fermentas, MA, USA). In each experiment, two RNA samples without reverse transcriptase were used as negative controls for DNA contamination. Real-time PCR used 2 µl of 1:5 diluted reverse transcriptase. Real-time PCR reactions were performed in a mixture containing 140 ng of specific primers (Table 2) and 10 μl of SYBR green master mix (Life Technology, MA, USA) in a total volume of 20 μl under the following conditions: initial denaturation at 95 °C for 10 min, followed by 40 cycles of denaturing at 95 °C for 20 s, annealing at 59 °C for 30 s, and extension at 72 °C the reference gene was GAPDH. Statistical analysis for relative mRNA expression was performed by the Relative Expression Software Tool (REST) proposed by Pfaffl^61^. REST estimated the fold change and P-value (0.05). Each primer pair examined amplification from serially diluted cDNA. They generated a 5-point standard curve in triplicate. All PCR assays displayed efficiencies of between 1.8 and 2.0.

### Primer design for HRM, real-time PCR analysis, and stem-loop

MethylPrimer Express V1.0 (ABI) and Methprimer (http://www.urogene.org/methprimer/index.html) were used to design miR-335 and miR-145 primers. MethBLAST (http://www.medgen.ugent.be/methBLAST) was used to perform BLAST. Allele ID 6 was used to create PD-L1 primers. The NCBI Nucleotide Program (http://blast.nlm.nih.gov/) was used to determine each primer's specificity. Chen et al.'s were changed to boost stem-loop flexibility and sensitivity. Adding 14 nucleotides to the original sequence lengthened the loop and enabled the design of a universal reverse primer and TaqMan probe inside it. Substitutions decreased the melting temperature of the stem section. This newly designed structure detects each miR by adding complimentary nucleotides to each stem-loop. Real-time PCR primers employed practically complete sequences of miR-335 and miR-145 (Table 2). Referencing U6.

### DNA Extraction and Bisulfite Modification

Per the manufacturer's instructions, genomic DNA was isolated from BC tissues and adjacent non-tumor tissues using the Universal Genomic DNA Extraction Kit (Qiagen, Germany). The DNA from these tissues was tested for quality and integrity using electrophoresis on a 1% agarose gel and quantified spectrophotometrically. Genomic DNA (2 µg) was then subjected to bisulfite conversion using an EZ DNA Methylation Kit (Qiagen, Germany). The bisulfite conversion reaction was incubated in a PCR thermocycler at 98 °C for 5 min, followed by 60 °C for 25 min, 95 °C for 5 min, 60 °C for 85 min, 95 °C for 5 min, and 60 °C for 175 min, with a final incubation at 4 °C for up to 20 h. The modified DNA samples were dissolved in ddH2O and stored at − 20 °C.

### Methylation studies by high-resolution melting analysis

Bisulfite-modified DNA was amplified using primers that targeted methylated regions of miR-335 and -145 promoters (Table 2). Diluting 100% methylated DNA with unmethylated bisulfite-modified DNA produced 25%, 50%, and 75% standard controls (Qiagen, Hilden, Germany). Each experiment had these standards. The reaction mixture contained 10 μl of Meltdoctor master mix, 10 ng of bisulfite-treated DNA, 200 nM of each primer, and 20 μl of PCR-grade water. Step one PCR and high-resolution melting (HRM) were performed (ABI)^62^.

All reactions were carried out at 95 °C for 15 min, 60 °C for 30 s, 72 °C for 20 s, and 65–90 °C at 0.1 °C/s (ramps). Step One: The program performed HRM-curve analysis, then compared fluorescence at the melting point to unmethylated DNA fluorescence. Triplicate experiments were done. After the fluorescence drop, melting curves were normalized using HRM software.

### MicroRNA transfections

Pre-miR precursors of pre-miR-335, pre-miR-145, and control pre-miR precursor (scramble) from Ambion (Life Technology, AM17102). Each well of a 12-well plate was seeded with 0.8 × 10^6^ cells one day before transfection. When the confluence of seeded cells reached 80%, they were transfected with 100 nM of synthetic pre-miR-335 or scramble oligonucleotides using HiPerFect (Qiagen, Germany), according to the company protocol. After six hours, the transfection medium was replaced with a fresh medium containing 10% FBS. Transfected cell lines were harvested at 24, 48, and 72 h post-transfection.

### Flow cytometry using cultured cell lines

For flow cytometric analysis, cells were first incubated with Fc receptor block (BD Biosciences, for human cells) or anti-CD16/32 (eBioscience, for mouse cells) with a LIVE/DEAD viability dye (Thermo Fisher Scientific) for 10 min at 4 °C in FACS buffer (PBS/0.5% BSA/2 mM EDTA), followed by staining with antibody panels for 30 min at 4 °C. The following human antibodies with corresponding isotype controls were used: the PD-L1 monoclonal antibody (Cat. No. 329705) was purchased from BioLegend. The results of flow cytometry were analyzed using FlowJo software.

### Western blot

Total protein was extracted from transfected and non-transfected cells using RIPA buffer (Beyotime, China) containing a protease inhibitor cocktail (Roche, USA). Protein concentration was quantified by a BCA protein assay kit (Beyotime, China). Lysates were combined with equal volumes of 2× Laemmli sample buffer. After 5 minutes of boiling, 15 μg of lysates were SDS-PAGEd and transferred to a 0.2 μm immune-BlotTM PVDF membrane (Cat. No. 162-017777; Bio-Rad Laboratories, CA, USA). 5% BSA (Cat. No. A-7888; Sigma Aldrich, MO, USA) in 0.1% Tween 20 blocked the membranes for 1 h. The primary antibodies for Western blotting were anti-PD-L1 (Cat. No. ab233482; Abcam) and anti-GAPDH (Cat. No. ab9485; Abcam). After the washing step, the membrane was incubated with secondary antibodies (goat anti-rabbit IgG H&L (HRP) (Cat No. ab6721; Abcam). Then, an ECL chromogenic substrate (BIO-RAD, USA) was applied to detect the signals. Densitometry of protein bands was done using Quantity One software to divide the percentage area under the curve of each band by its corresponding GAPDH band, and then calculated values were compared between them.

### Cell proliferation analysis

Cell proliferation was assessed using the MTT kit (Sigma, St. Louis, MO, USA). Briefly, MDA-MB231, BT549, and MCF7 (3 × 10^4^ cells/well in 96-well microplates) were transfected with pre-miR-335, miR-145, or negative control. Cells in the logarithmic growth phase were harvested and seeded on a 96-well plate. At 24, 48, and 72 h after seeding, 10 μl of MTT was added to each well, and the cells were incubated for 4 h. We supplemented each well with 150 μl of DMSO, and the optical density (OD) was recorded at 540 nm. Viability = 100 × (absorbance of the treated sample) / (absorbance of control). All experiments were repeated three times, and the average results were calculated. The experiments were repeated three times, and average results were calculated.

### Cell cycle and apoptosis assay

To determine the effects of miR-335 and -145 on apoptosis and cell cycle in BC cells, MDA-MB231, BT549, and MCF7 cells were seeded in 24-well plates with a density of 1 × 10^6^ cells/mL in growth medium one day before transfection. The cells were transfected with scramble oligonucleotides or pre-miR-335 and pre-miR-145 by HiPerFect (Qiagen, Germany) according to the manufacturer's protocol. A cold 70% ethanol solution was applied to the cells for 24 hours at − 20 °C. The cells were stained with a propidium iodide (PI) solution after a washing step and left to dry for 30 min at room temperature. Flow cytometry was used to analyze the cell cycle (BD Biosciences, San Jose, CA, USA). According to the manufacturer's instructions, cells were stained twice with FITC-labeled Annexin V and PI using the Annexin V-FITC/PI Apoptosis Detection Kit (Solarbio, CA1020). We repeated this test three times, and the data it produced was analyzed by flow cytometry in FL1 and FL3 channels in the Partec Flow cytometer (Supplementary file).

### Statistical analysis

At least three real-time PCR, cell proliferation, cell cycle, and apoptosis tests were run. The data is mean ± SEM. Each test's data was entered into GraphPad Prism V.8 for statistical analysis using one-way ANOVA. The t-test was used to compare cancerous and non-cancerous breast cells and samples. All real-time PCR results were analyzed using the CT technique with REST 2009 software (Qiagen, Hilden, Germany) and normalized against GAPDH for mRNA and U6 for miRNA. Pearson's correlation coefficient tested the two groups' correlation. P < 0.05 signifies statistical significance. Log-rank was used to compare Kaplan–Meier survival curves.

### Ethics approval and consent to participate

The present study was conducted under the instructions accepted by the Ethics Committee of Pasteur Institute of Iran (Ethical code: IR.PII.REC.1398.023), written informed consent to participate, and consent to publish forms was obtained from all participants involved in the present study.

### Informed consent

Written informed consent was obtained from all enrolled subjects.

## Supplementary Information

Supplementary Information.

## Data Availability

All data generated or analyzed during this study are included in this published article.

## Abbreviations

PD-L1: Programmed cell death ligand 1
BC: Breast cancer
miR-335: MicroRNA-335-3p
miR-145: MicroRNA-145-5p
HRM: High Resolute Melting
PI: Propidium iodide
FDA: Food and Drug Administration
