# Supplementary material for: Aberrant promoter hypermethylation of miR-335 and miR-145 is involved in breast cancer PD-L1 overexpression
Source: Sci Rep. 2023 Jan 18;13:1003. doi: 10.1038/s41598-023-27415-8 (PMC9849328; doi:10.1038/s41598-023-27415-8)

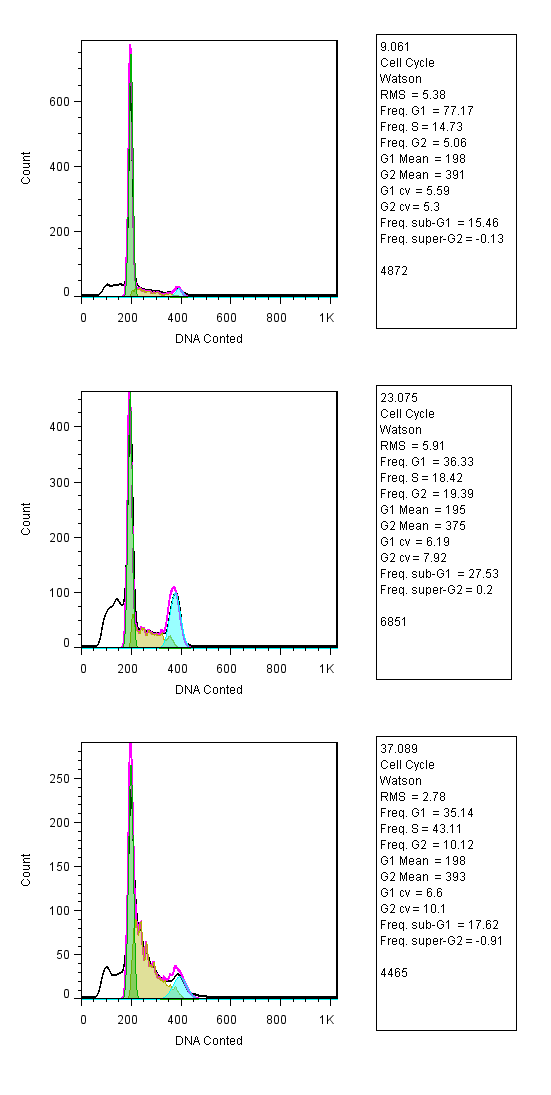

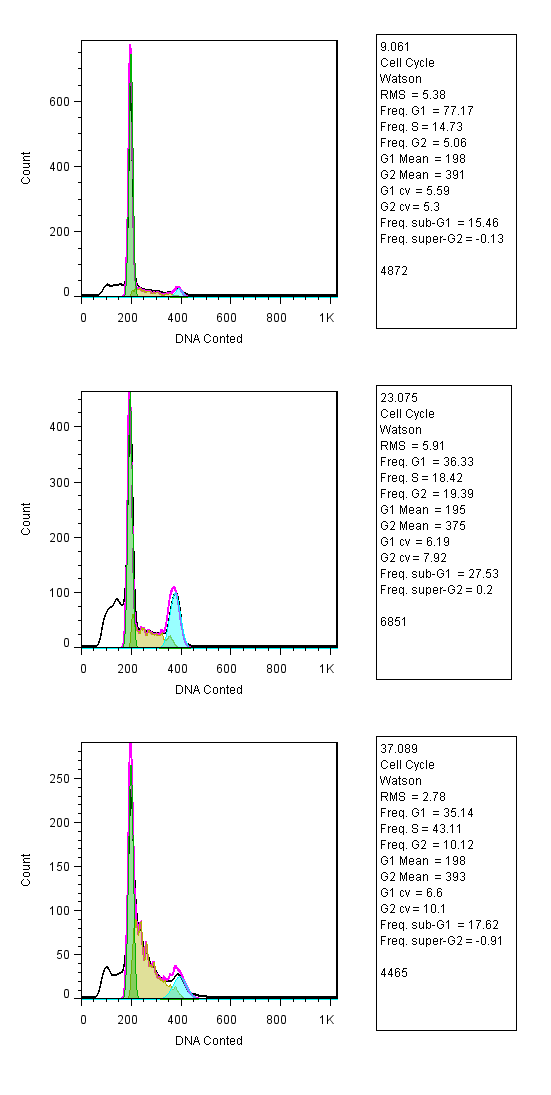

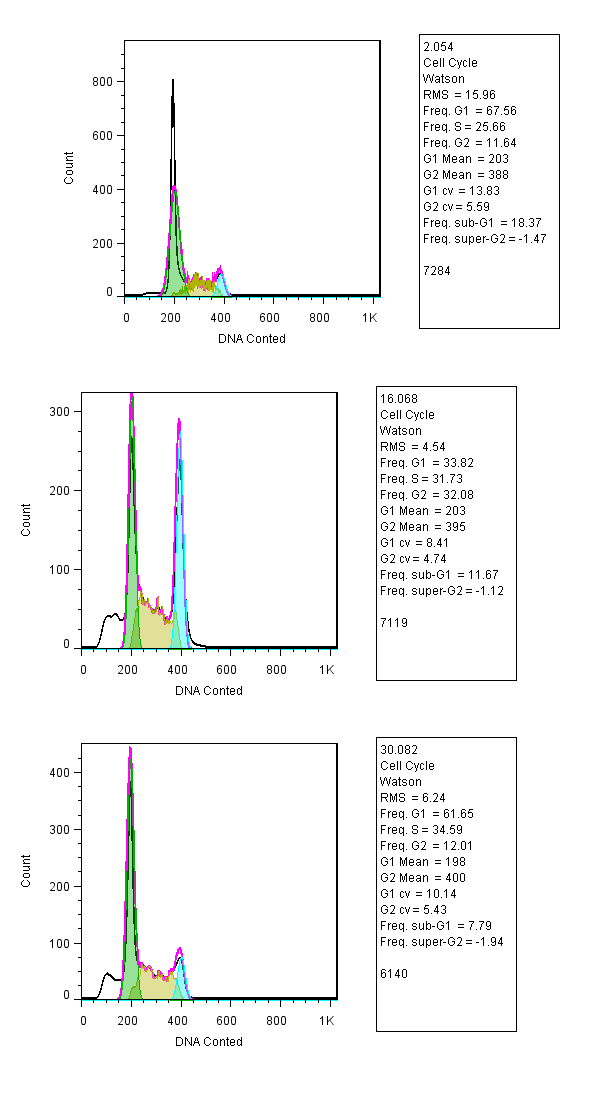

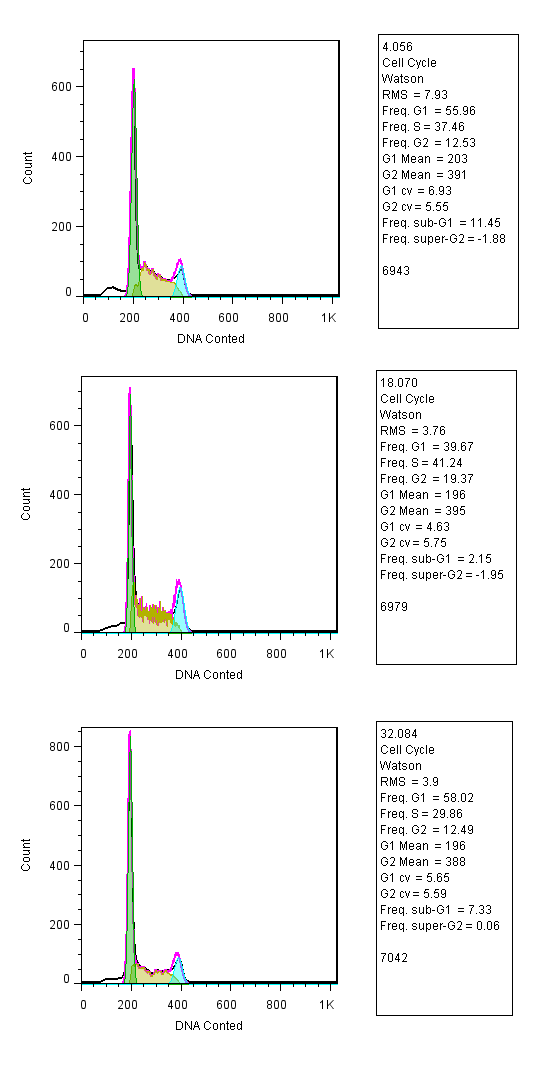

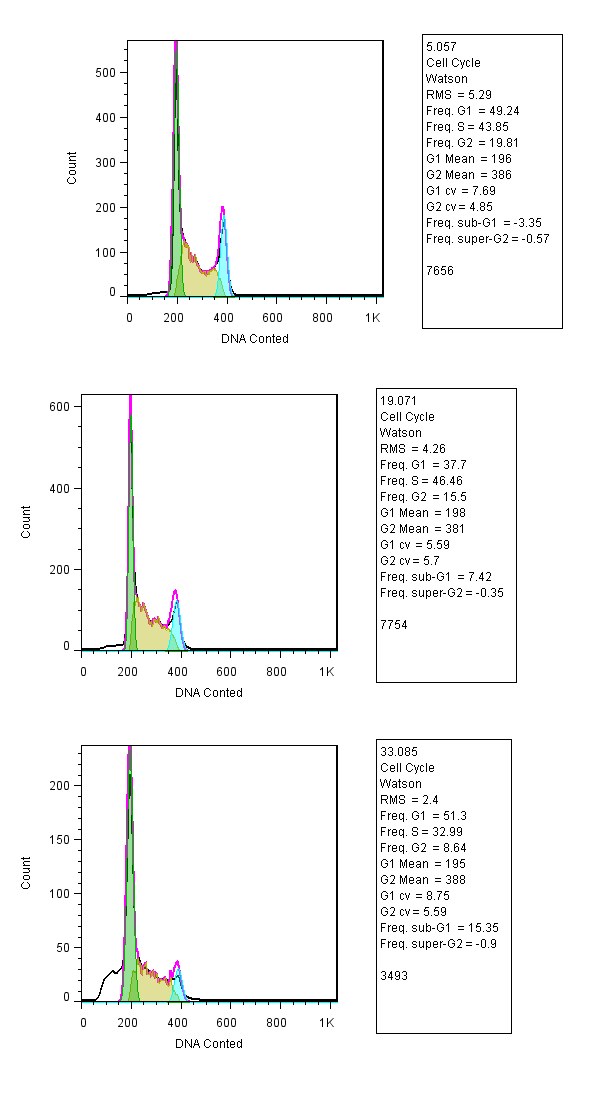

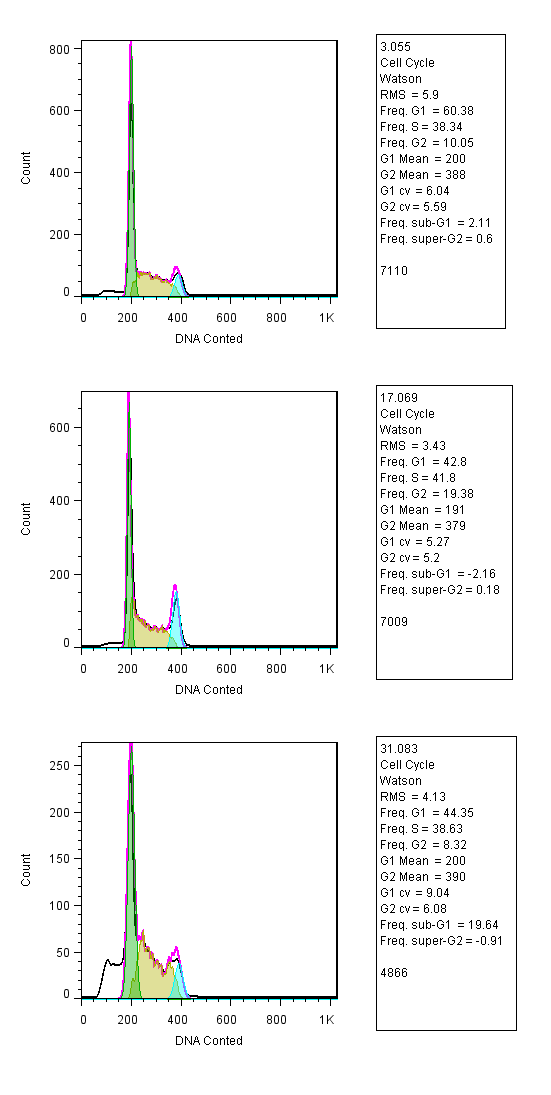

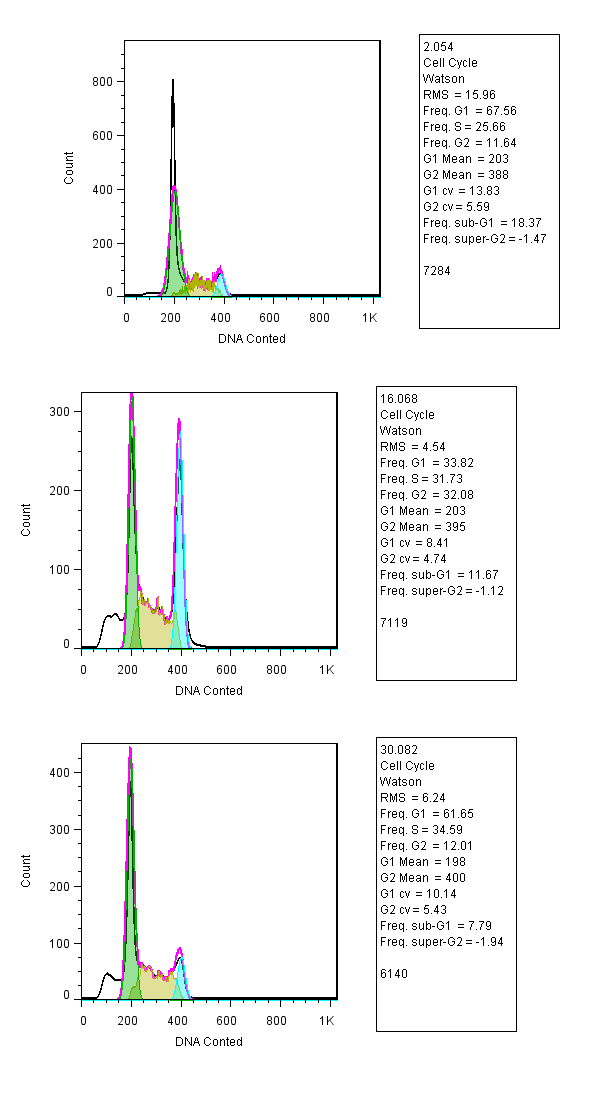

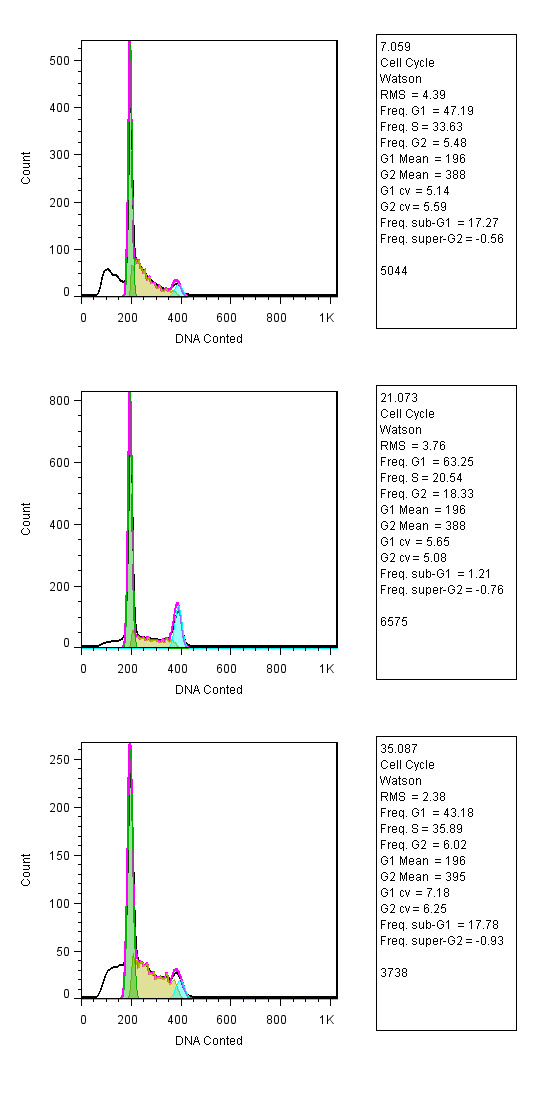

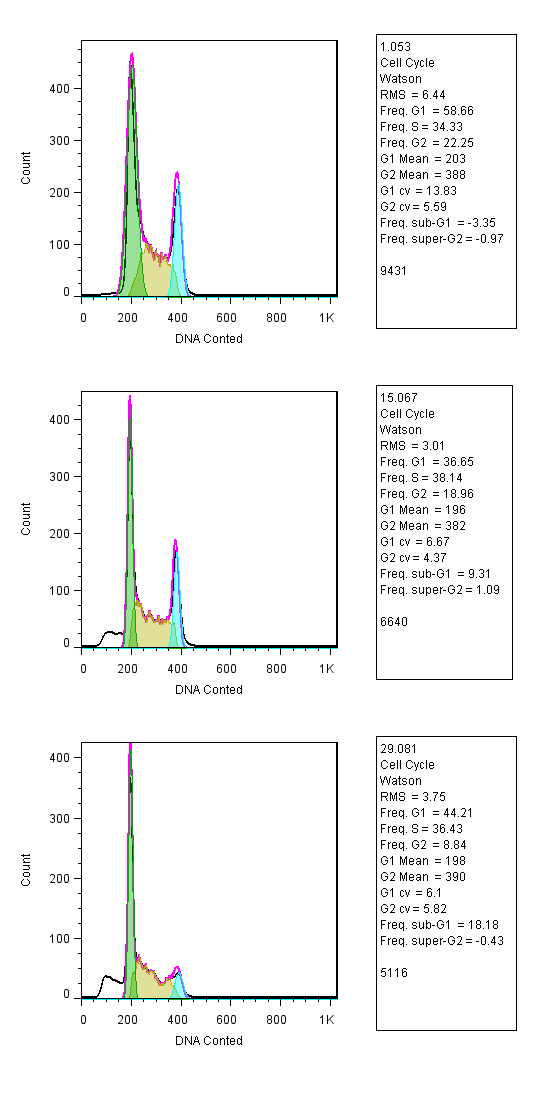

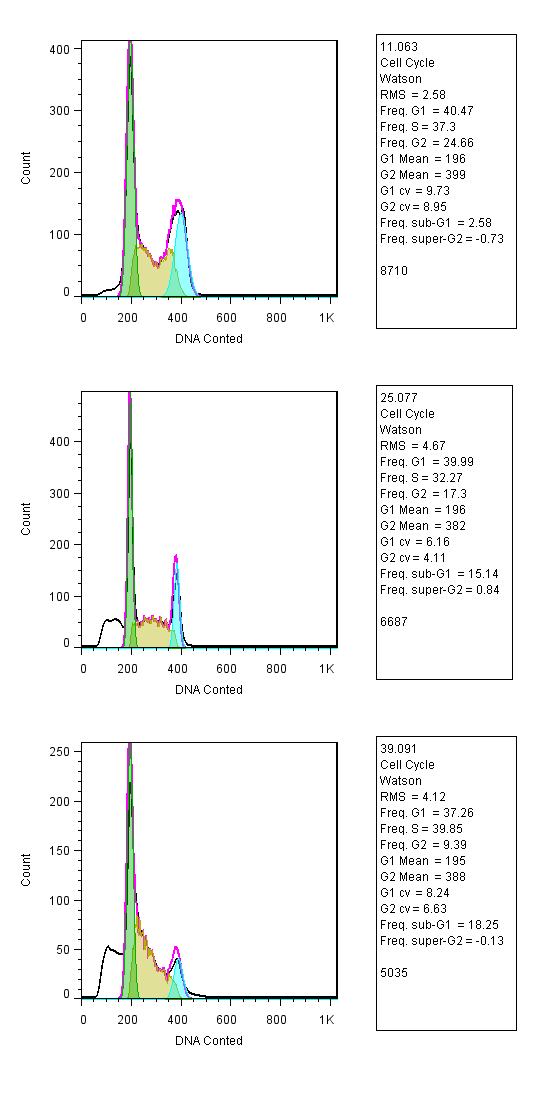

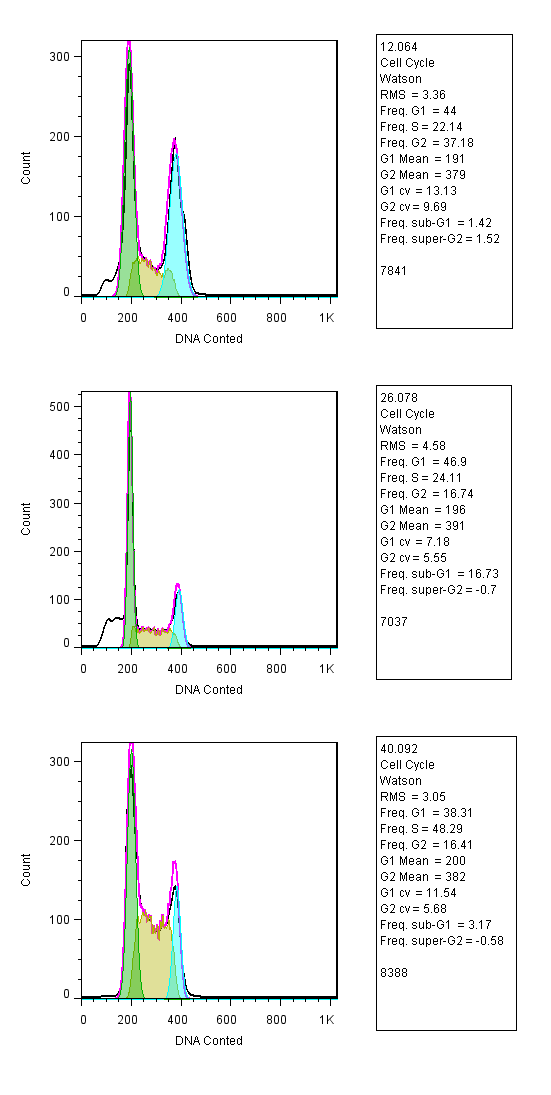

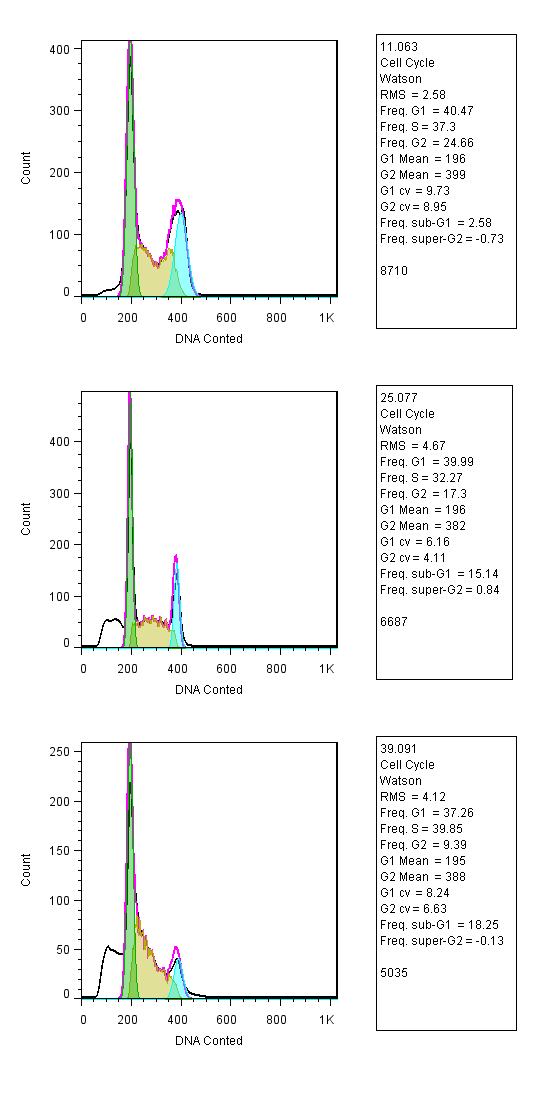

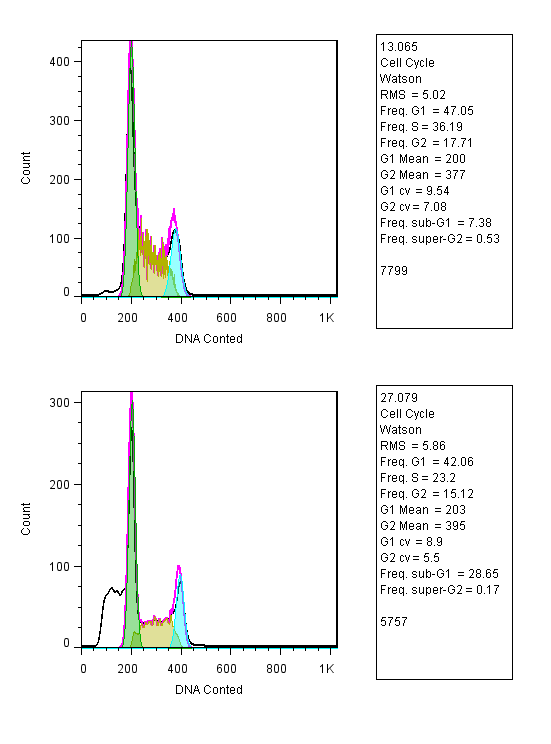

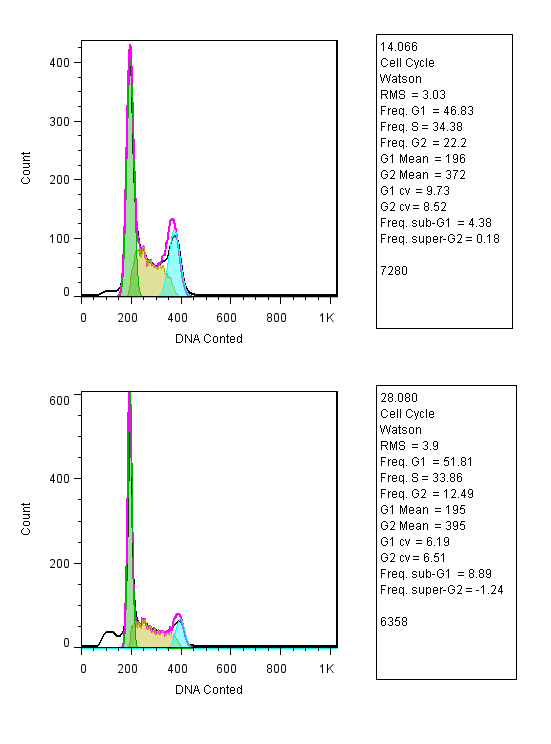

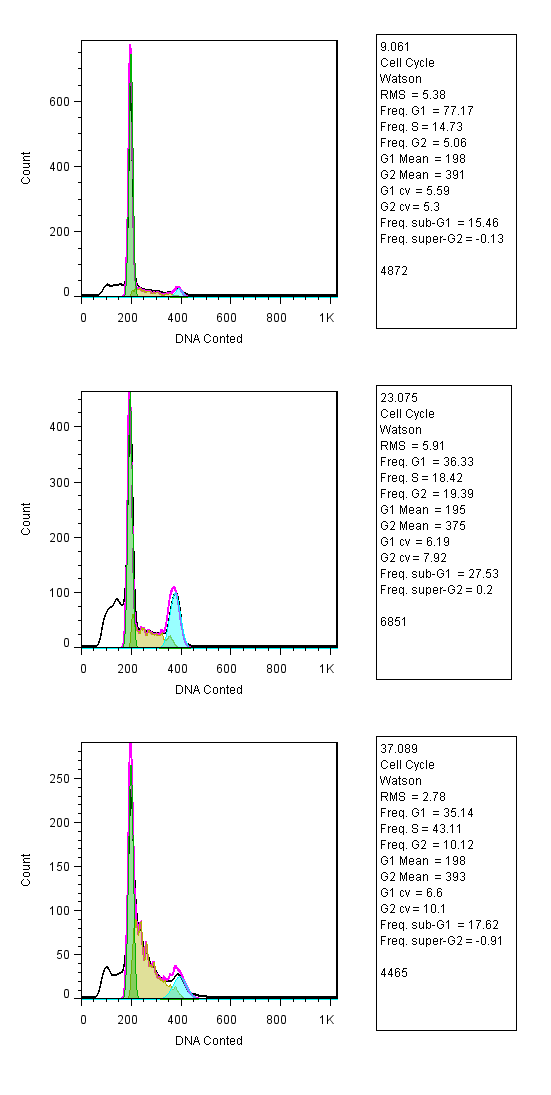

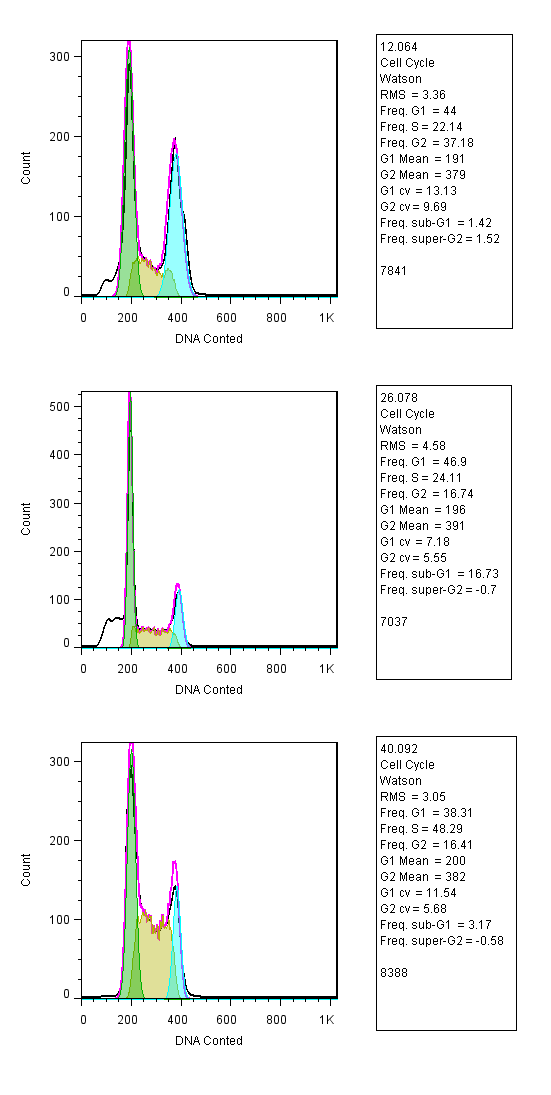

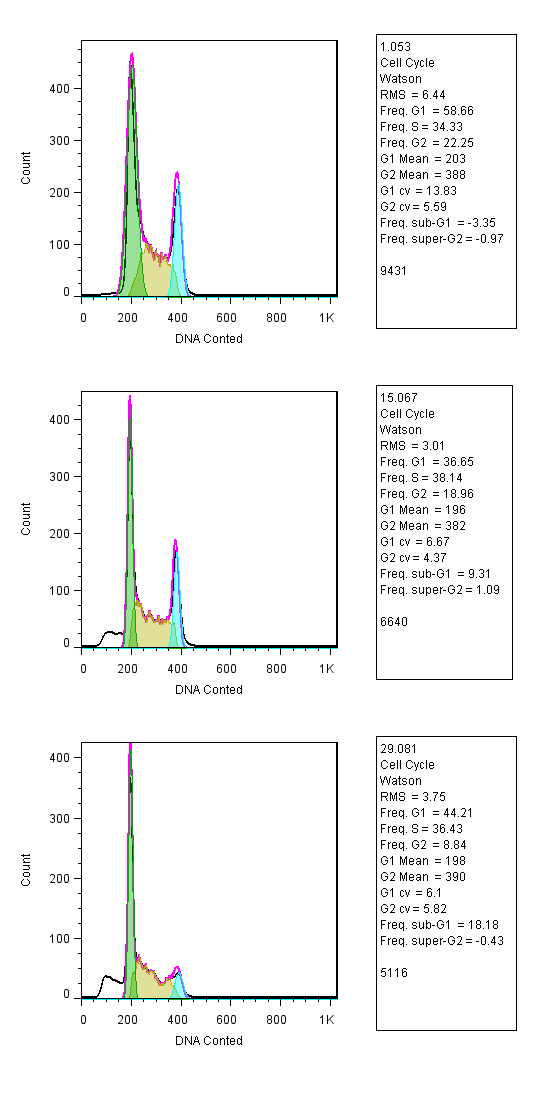

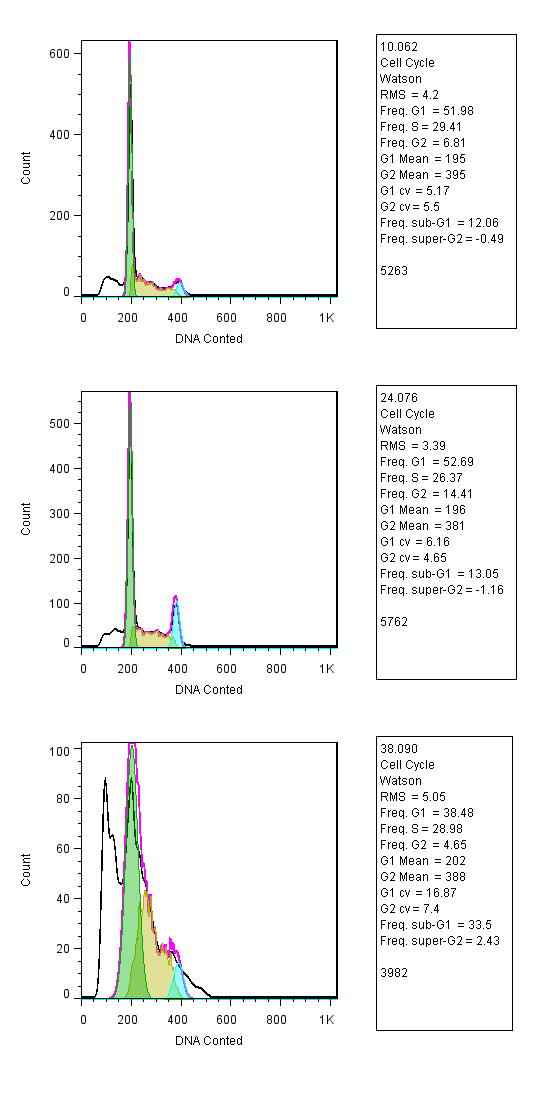

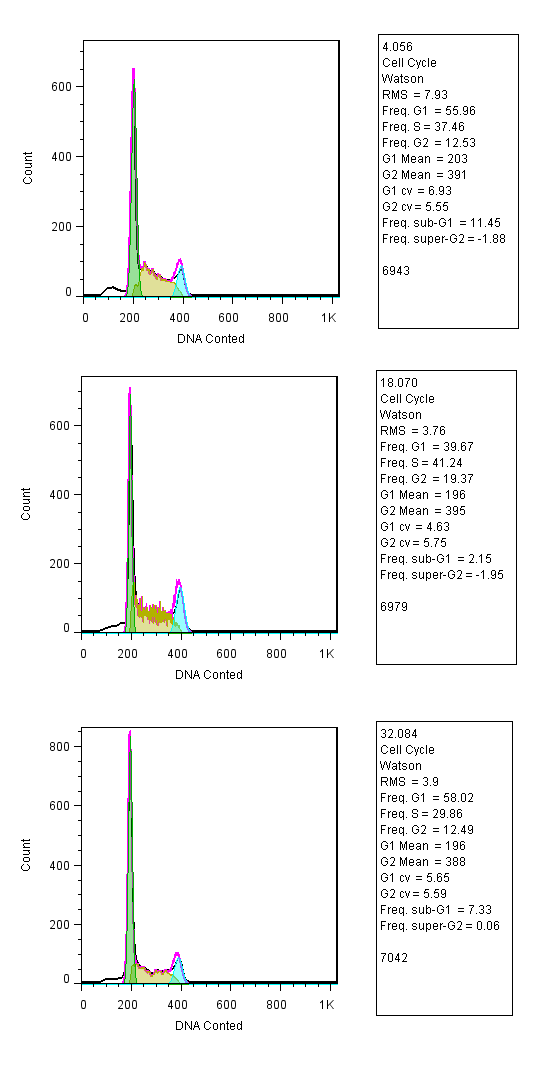

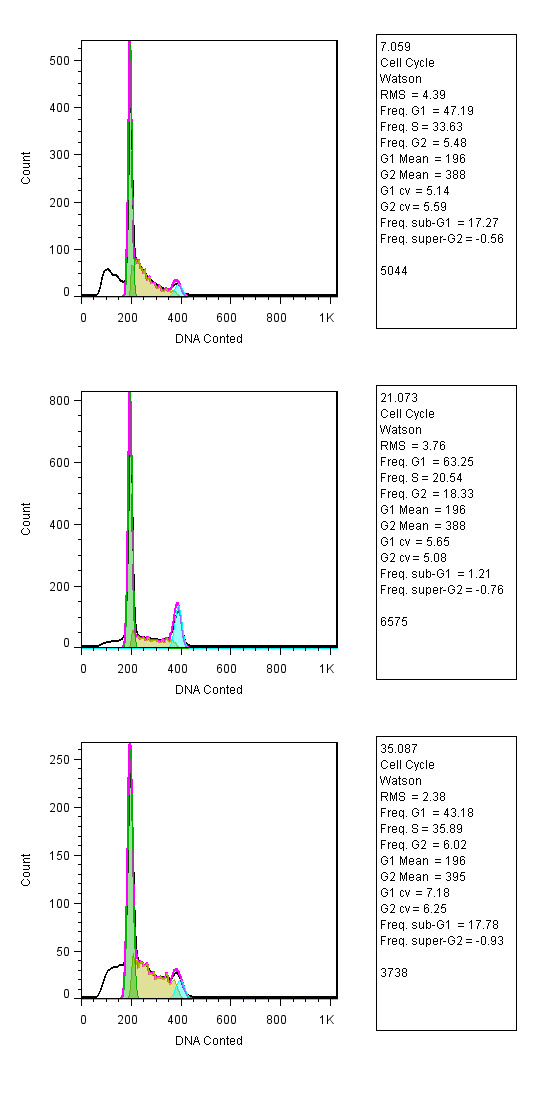

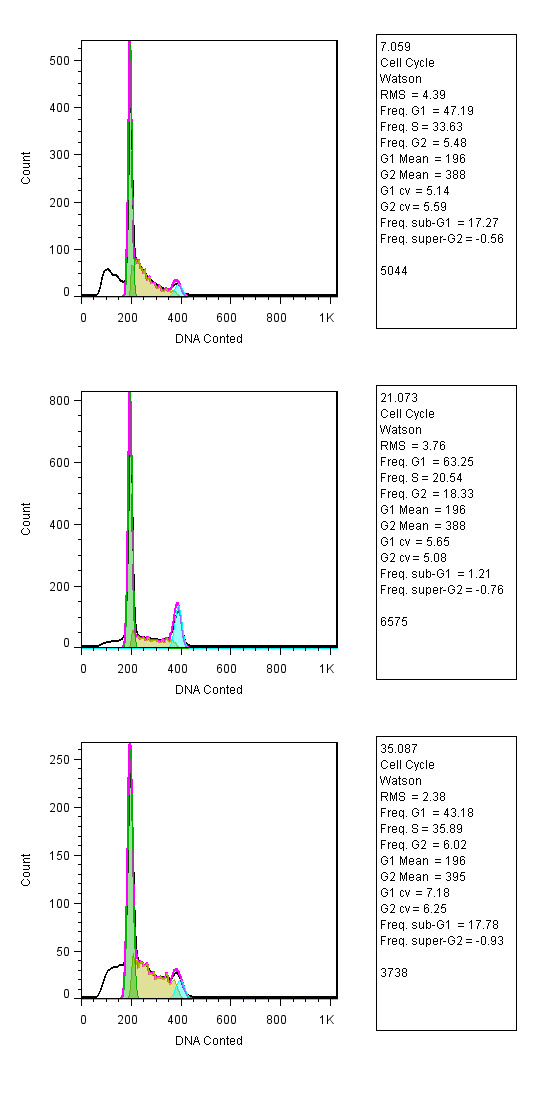

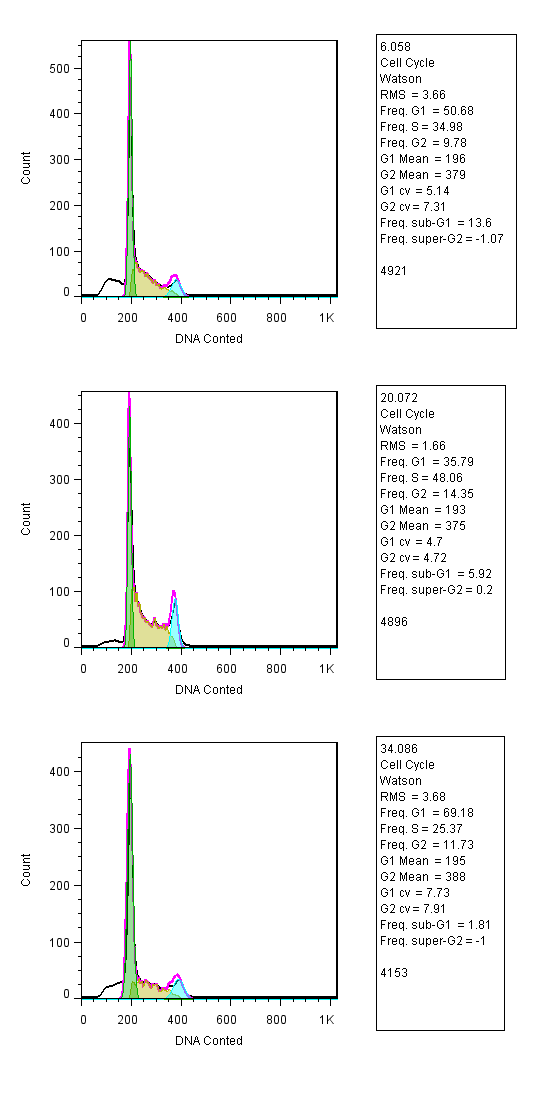

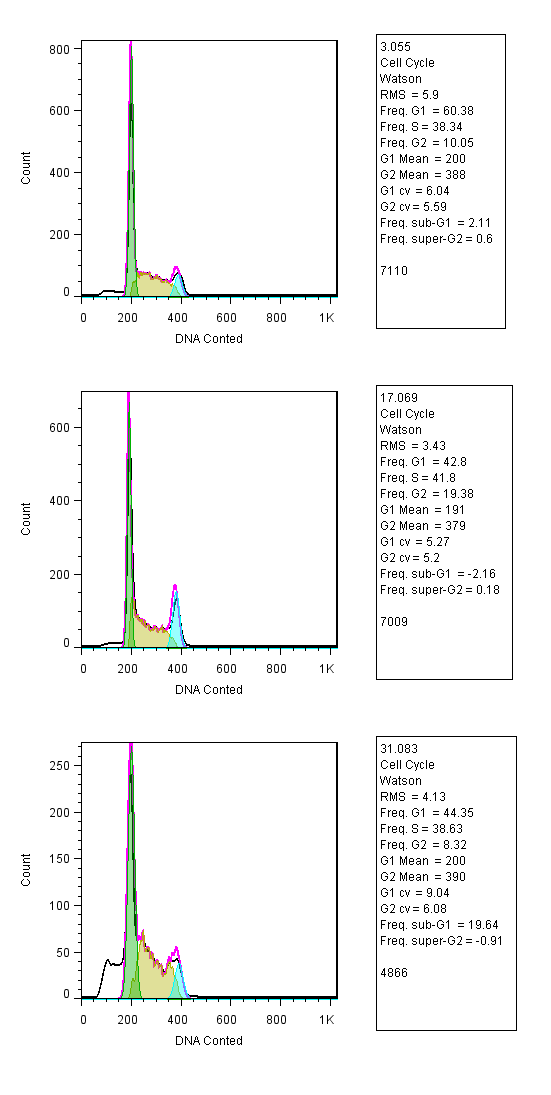

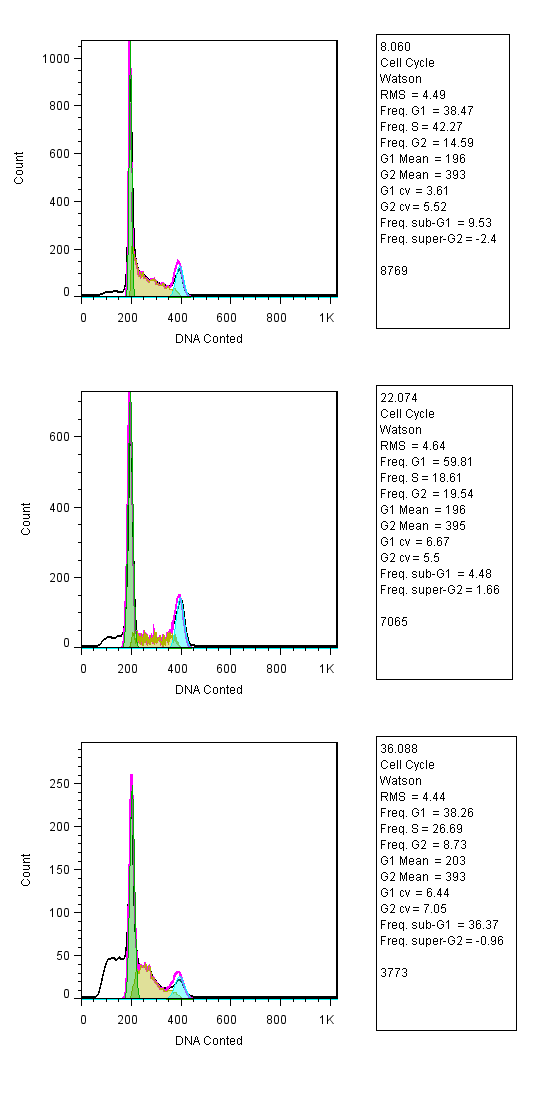


**Freq. G1: 47.19**

**Freq. S: 33.63**

**Freq. G2: 5.48**

**Freq. G1: 35.14**

**Freq. S: 43.11**

**Freq. G2: 10.12**

**Freq. G1: 77**

**Freq. S: 14.73**

**Freq. G2: 5.06**

**Freq. G1: 36.33**

**Freq. S: 18.42**

**Freq. G2: 19.39**

**Freq. G1: 44.21**

**Freq. S: 36.43**

**Freq. G2: 8.84**

**Freq. G1: 48.66**

**Freq. S: 34.33**

**Freq. G2: 25.25**

**Freq. G1: 46.83**

**Freq. S: 34.38**

**Freq. G2: 22.2**

**Freq. G1: 42.06**

**Freq. S: 23.2**

**Freq. G2: 15.12**

**Freq. G1: 46.9**

**Freq. S: 24.11**

**Freq. G2: 16.74**

**Freq. G1: 44**

**Freq. S: 22.14**

**Freq. G2: 37.18**

**Freq. G1: 37.26**

**Freq. S: 39.85**

**Freq. G2: 9.39**

**Freq. G1: 40.47**

**Freq. S: 37.3**

**Freq. G2: 24.66**

**Freq. G1: 52.69**

**Freq. S: 26.37**

**Freq. G2: 14.41**

**Freq. G1: 63.25**

**Freq. S: 20.54**

**Freq. G2: 18.33**

**Freq. G1: 47.19**

**Freq. S: 33.63**

**Freq. G2: 5.48**

**Freq. G1: 50.68**

**Freq. S: 34.98**

**Freq. G2: 9.78**

**DNA Count.**

**Events**

**MDA-MB231**

**BT549**

**MCF7**


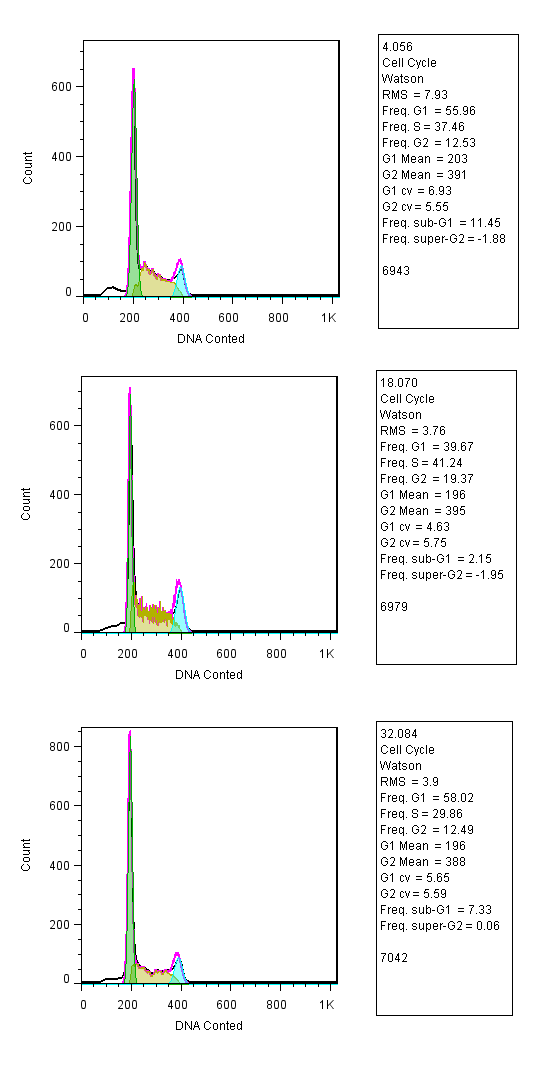

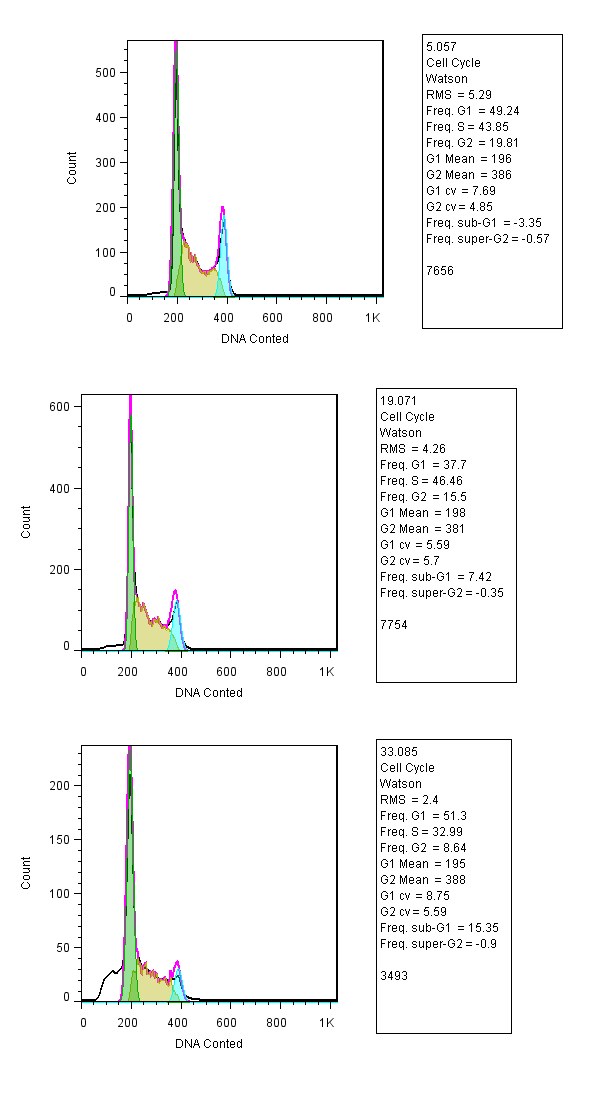

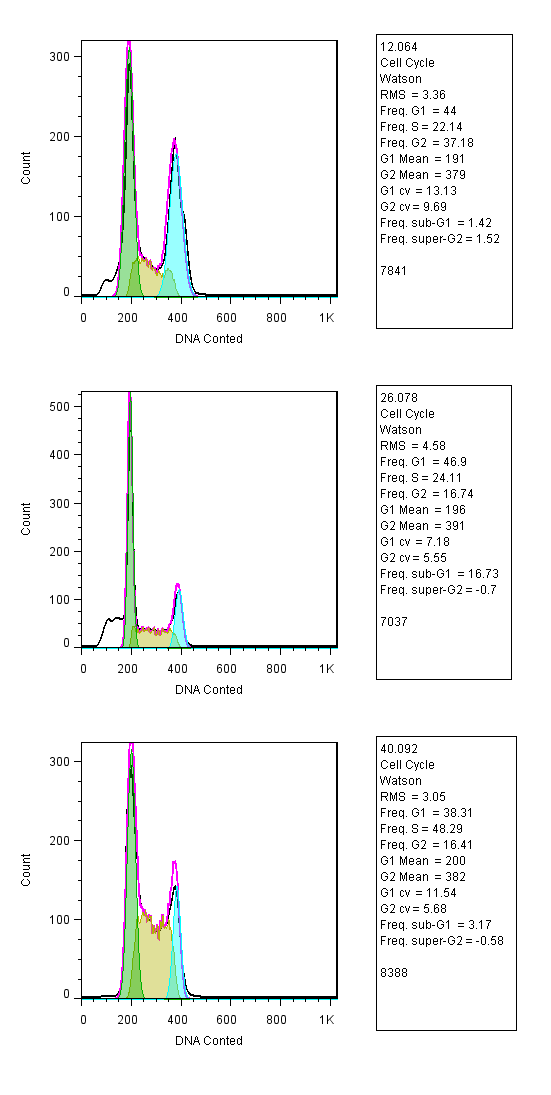

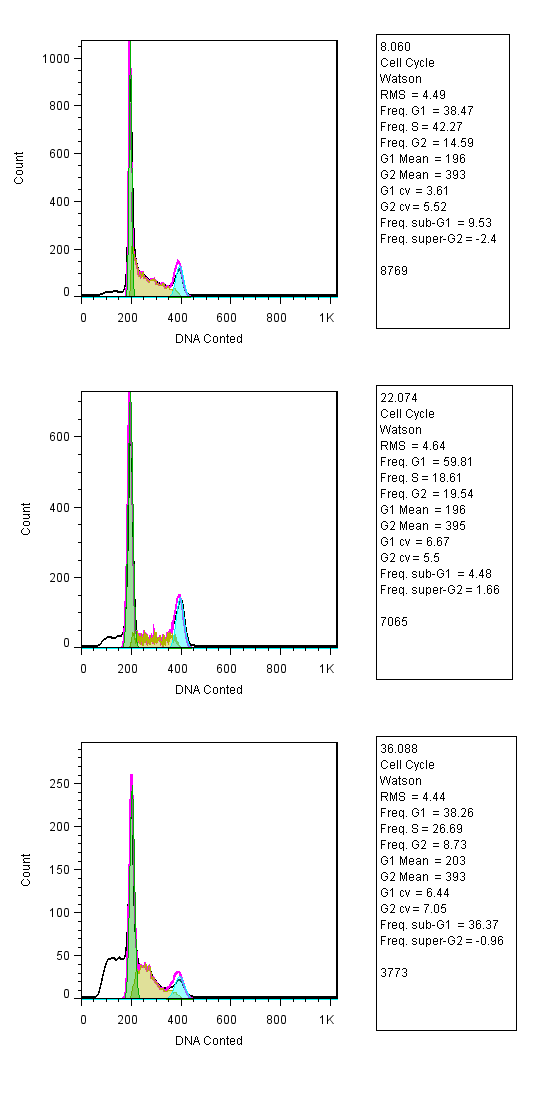

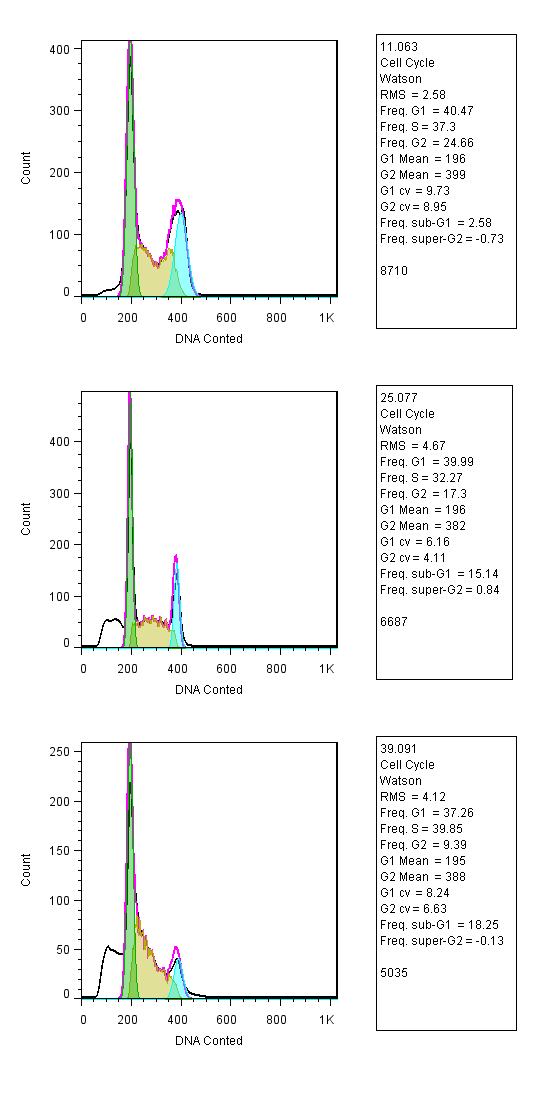

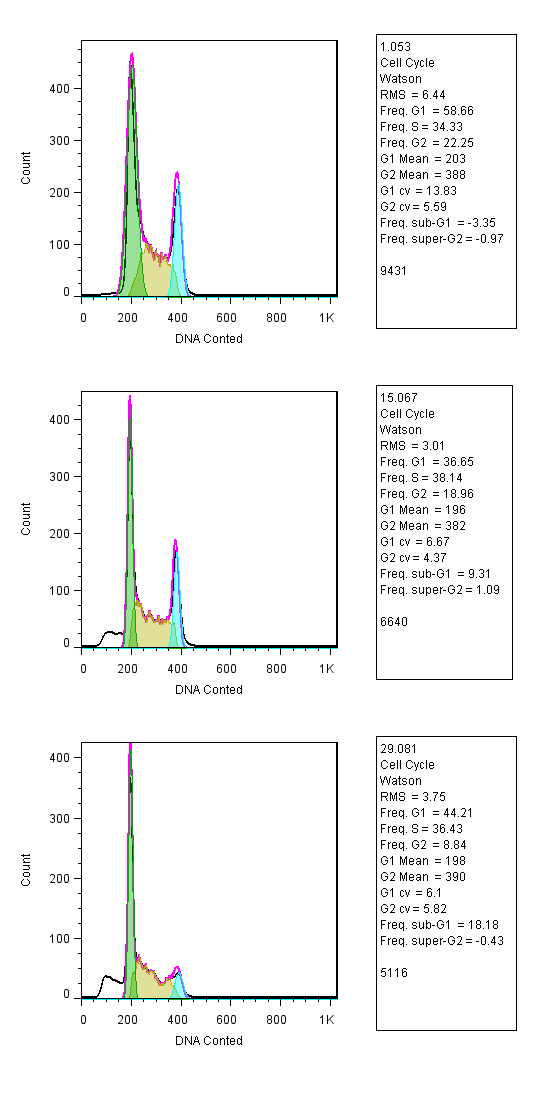

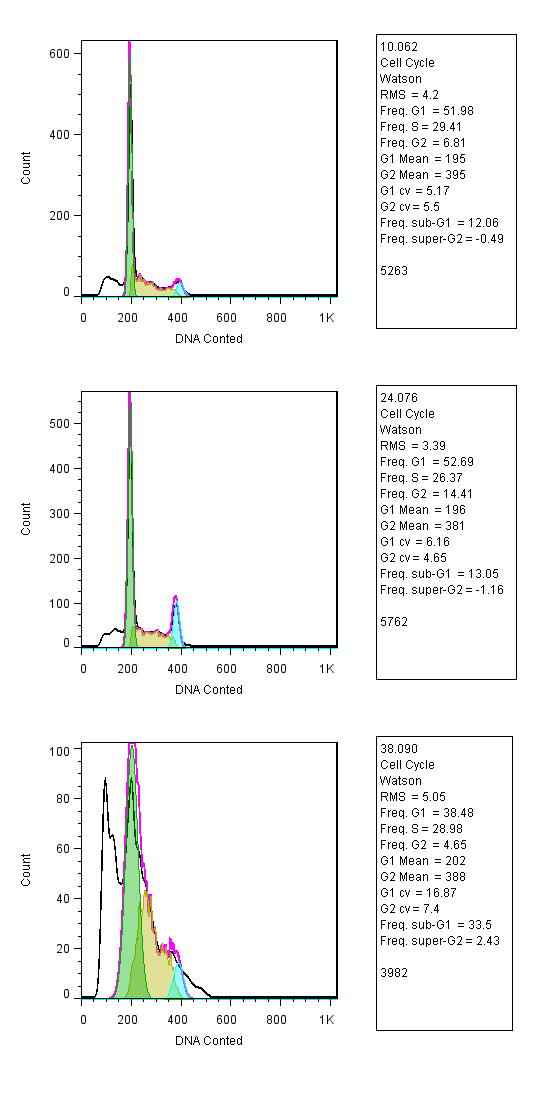

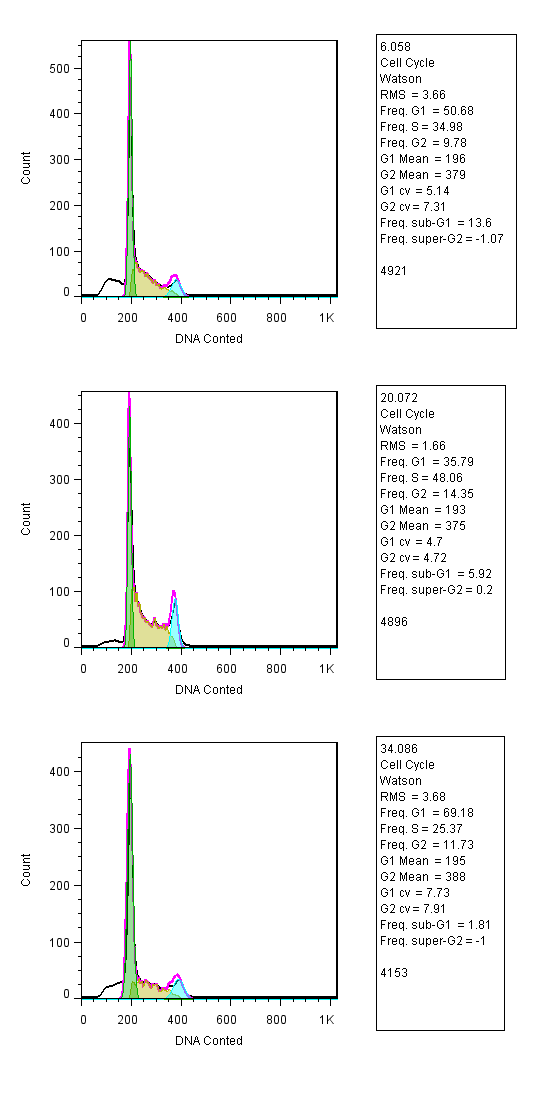

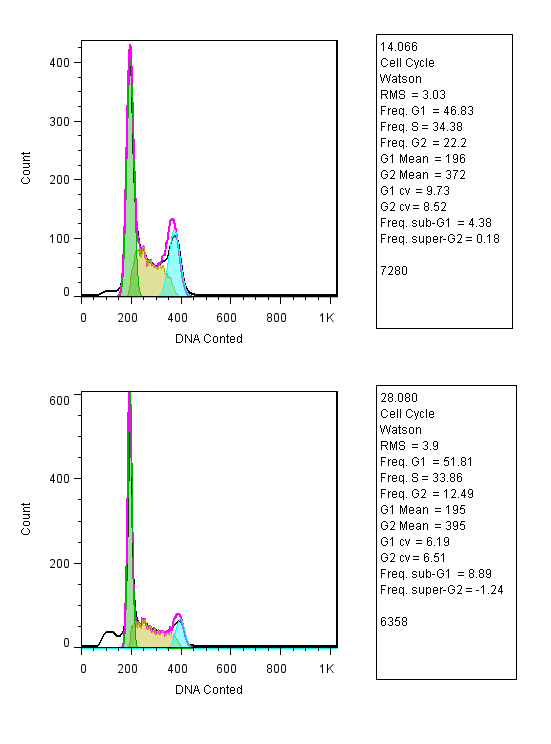

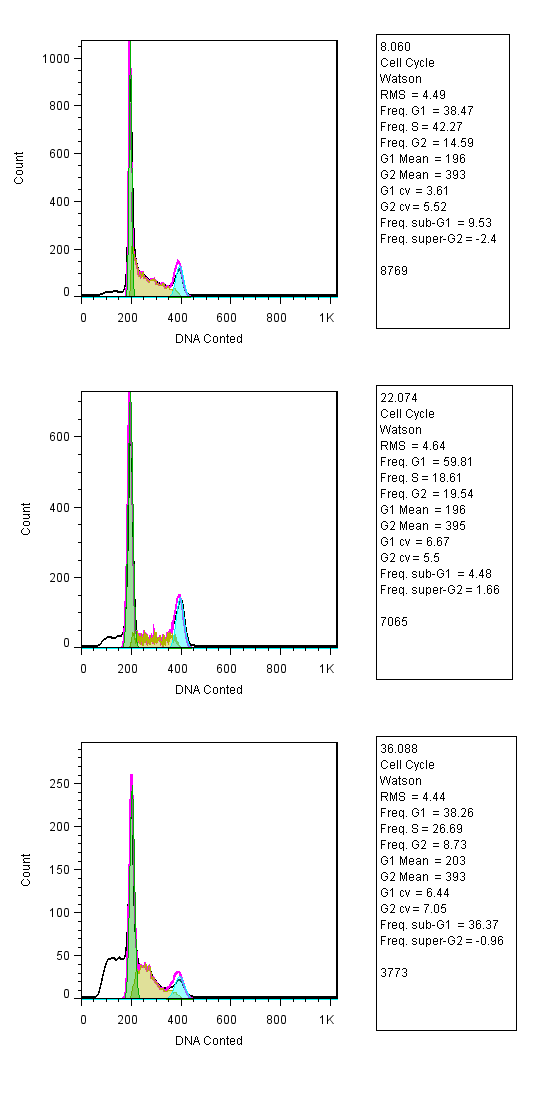

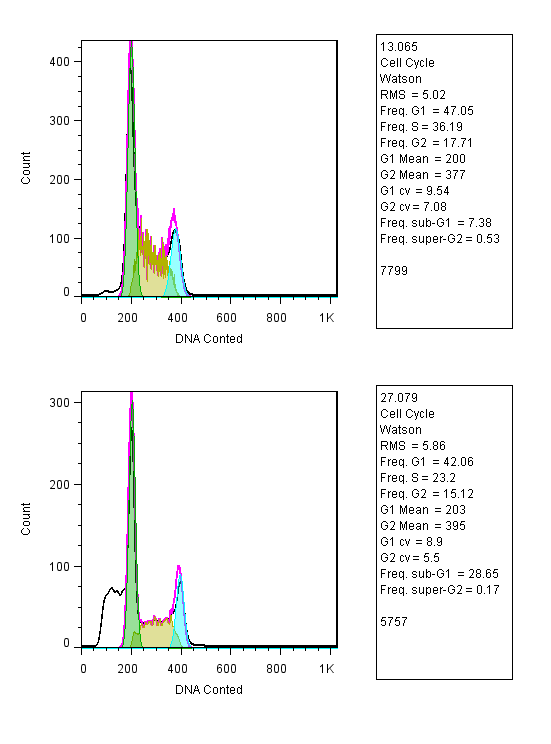


**Control**

**Scrambled**

**miR-335**

**miR-145**

**Freq. G1: 38.31**

**Freq. S: 48.29**

**Freq. G2: 16.41**

**Freq. G1: 38.47**

**Freq. S: 42.27**

**Freq. G2: 14.59**

**Freq. G1: 51.98**

**Freq. S: 29.41**

**Freq. G2: 6.81**

**Freq. G1: 59.61**

**Freq. S: 18.61**

**Freq. G2: 19.54**

**Freq. G1: 50.68**

**Freq. S: 34.98**

**Freq. G2: 9.78**

**Freq. G1: 47.05**

**Freq. S: 36.19**

**Freq. G2: 17.71**

**Freq. G1: 36.65**

**Freq. S: 38.14**

**Freq. G2: 18.96**

**Freq. G1: 39.67**

**Freq. S: 41.24**

**Freq. G2: 19.37**

**Freq. G1: 37.7**

**Freq. S: 46.46**

**Freq. G2: 15.5**

**Freq. G1: 39.99**

**Freq. S: 32.27**

**Freq. G2: 17.3**

**Freq. G1: 51.81**

**Freq. S: 33.86**

**Freq. G2: 12.49**


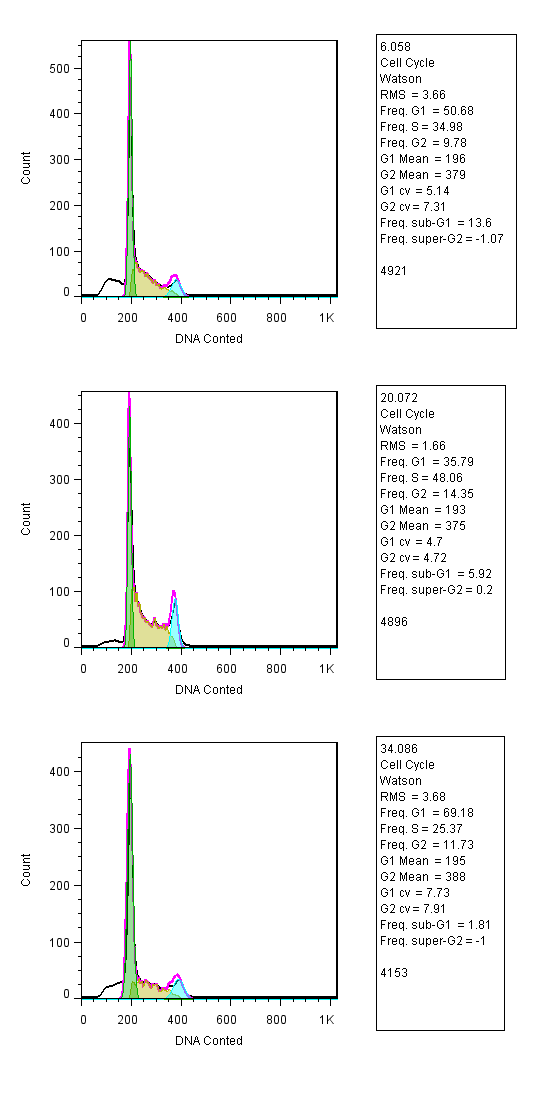


**Freq. G1: 69.18**

**Freq. S: 25.37**

**Freq. G2: 11.73**

**Freq. G1: 45.7**

**Freq. S: 43.41**

**Freq. G2: 16.5**

**Freq. G1: 58.02**

**Freq. S: 29.86**

**Freq. G2: 12.49**

**Freq. G1: 55.96**

**Freq. S: 37.46**

**Freq. G2: 12.53**

**Freq. G1: 42.8**

**Freq. S: 41.8**

**Freq. G2: 19.38**

**Freq. G1: 60.38**

**Freq. S: 38.34**

**Freq. G2: 10.05**

**Freq. G1: 61.65**

**Freq. S: 35**

**Freq. G2: 13.5**

**Freq. G1: 33.82**

**Freq. S: 31.73**

**Freq. G2: 32.08**

**Freq. G1: 38.26**

**Freq. S: 26.69**

**Freq. G2: 9.73**


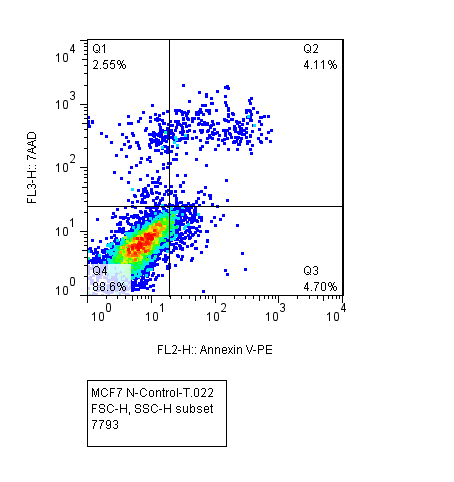

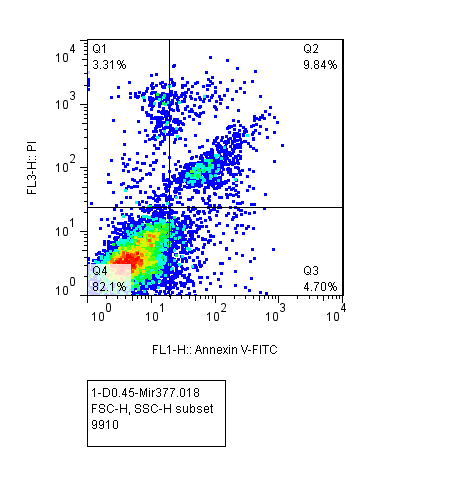

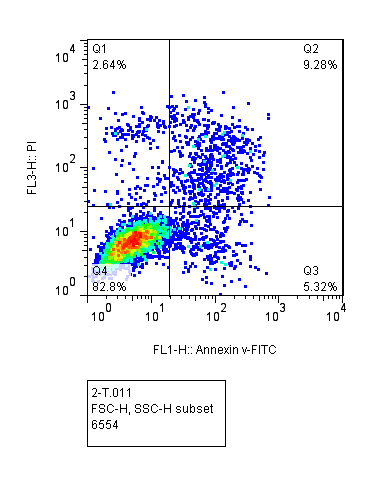

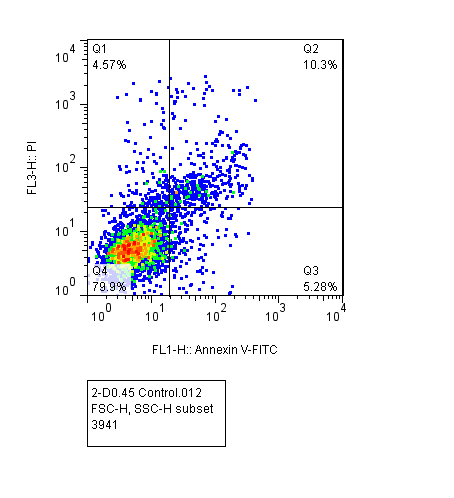

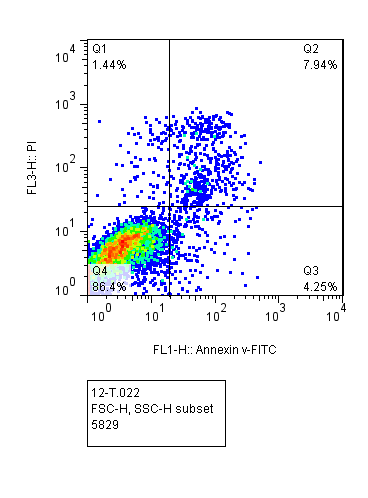

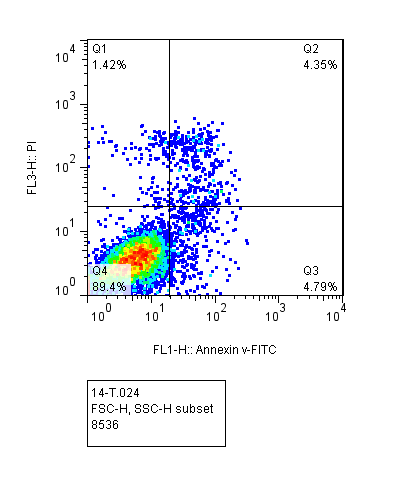

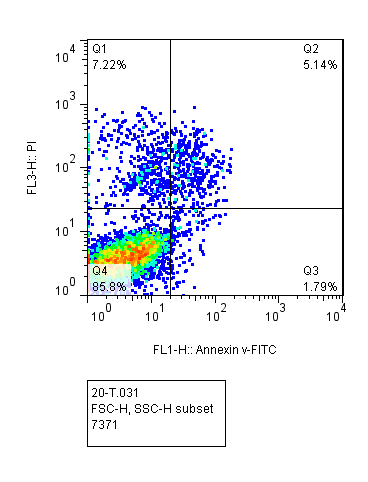

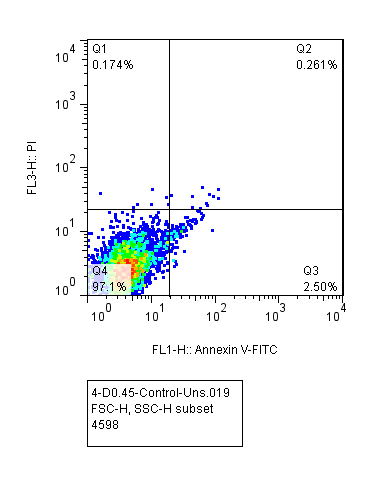

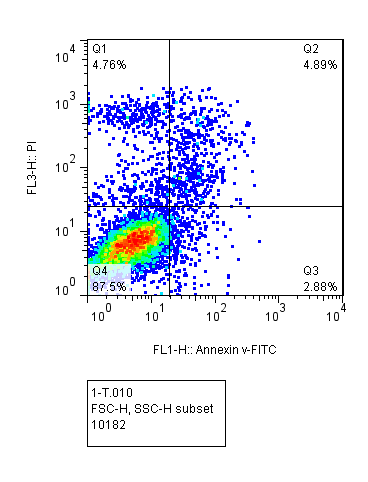

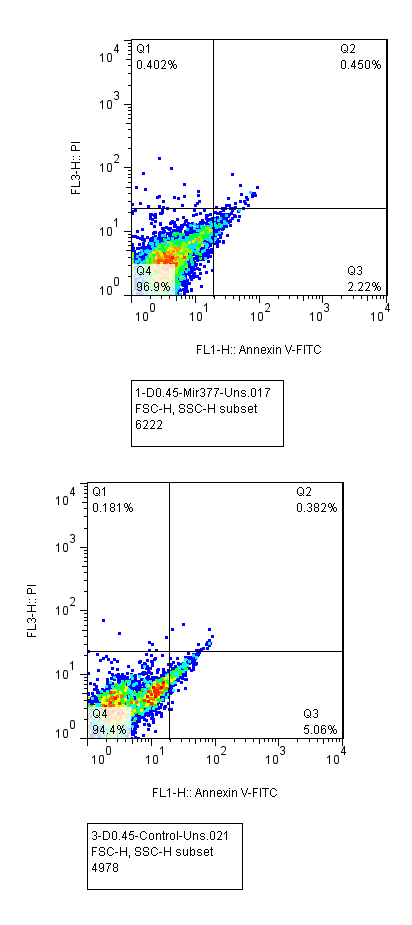

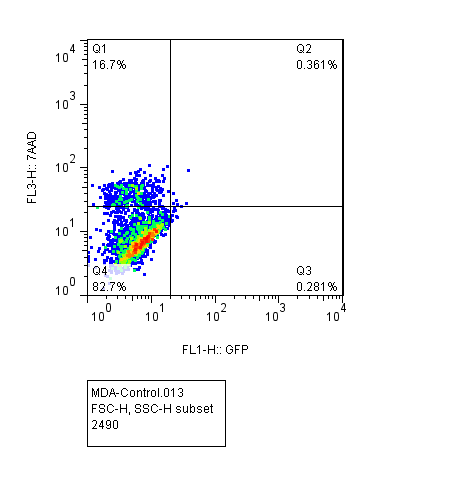

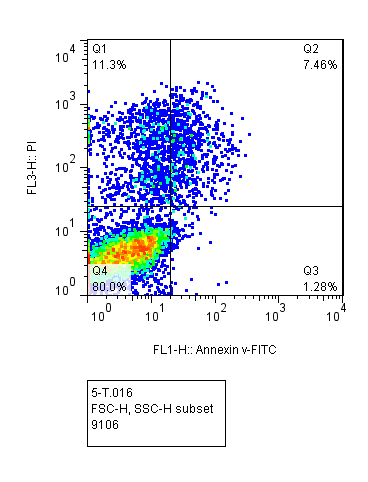

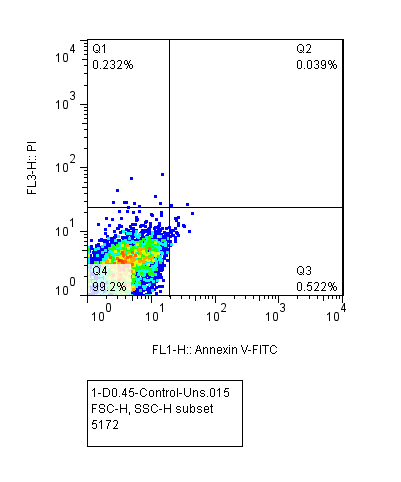

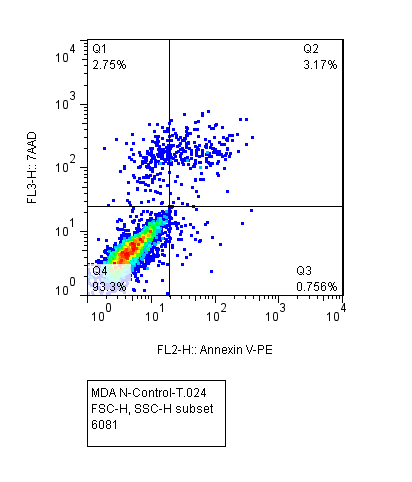

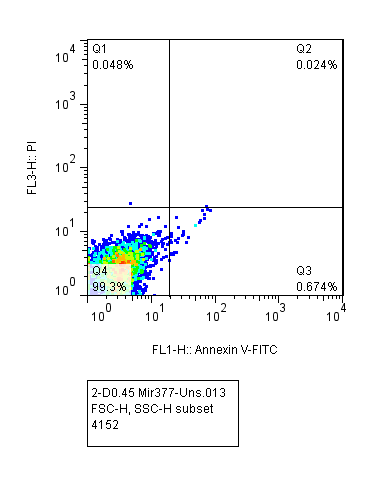

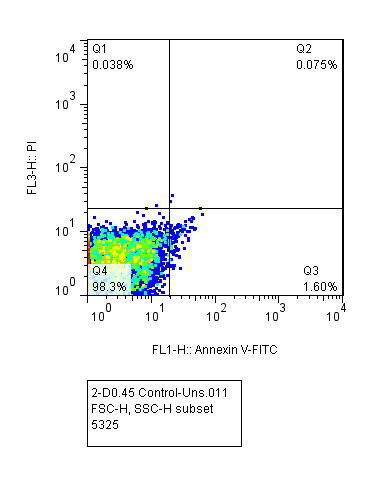

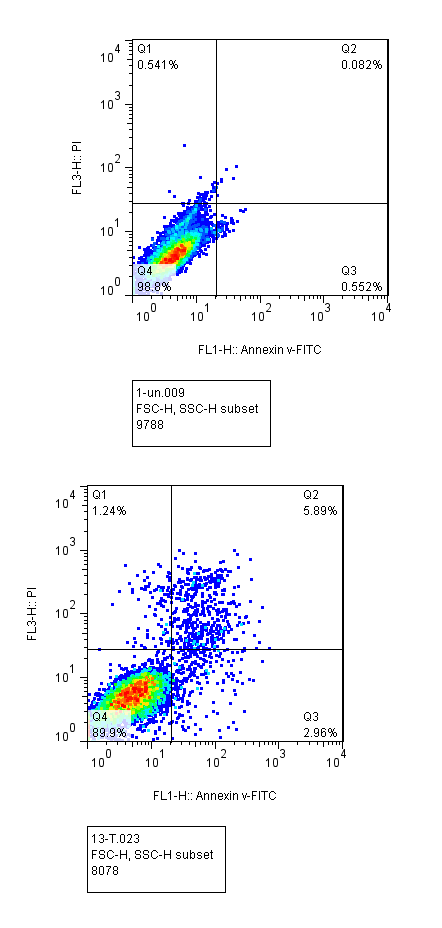


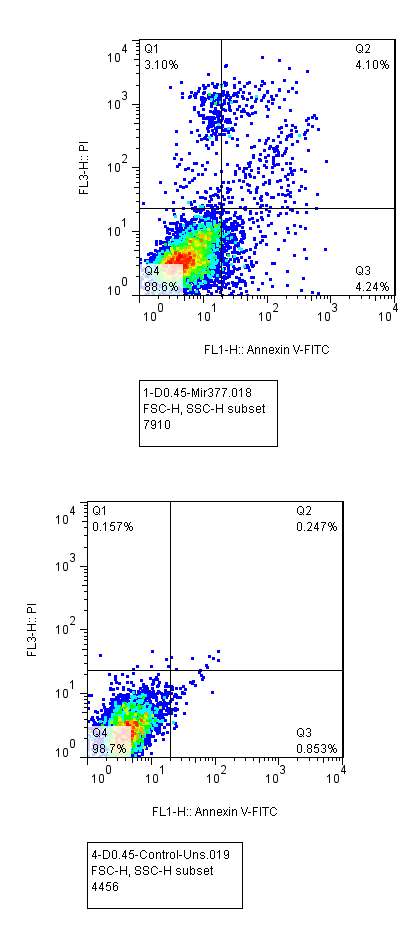

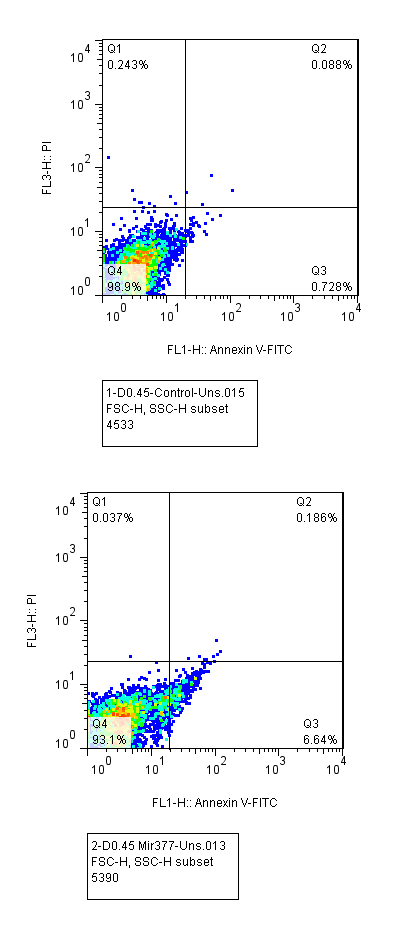

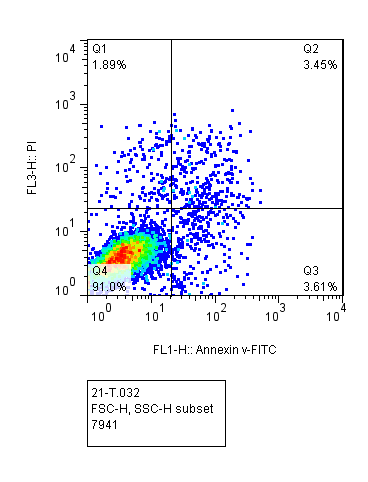

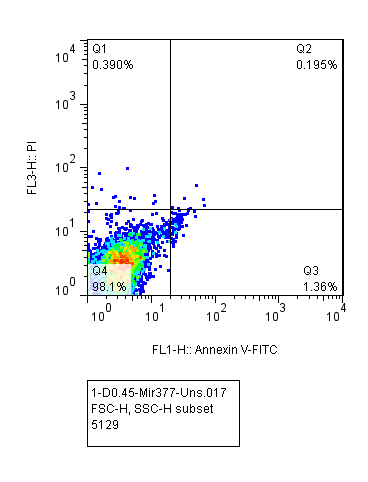

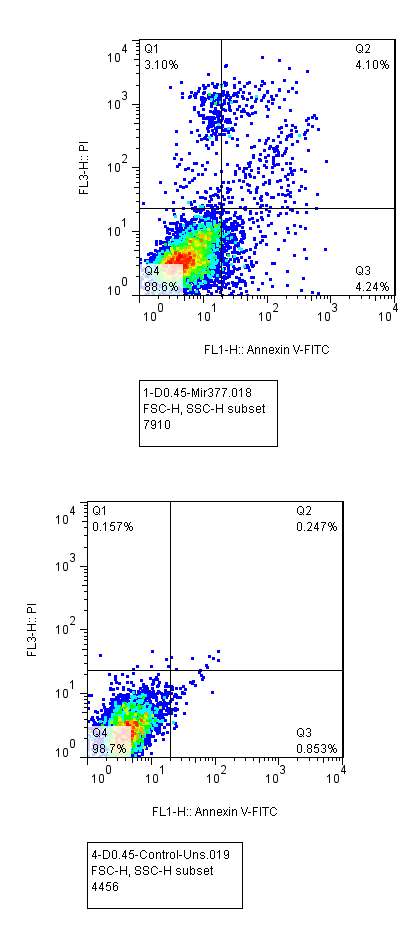

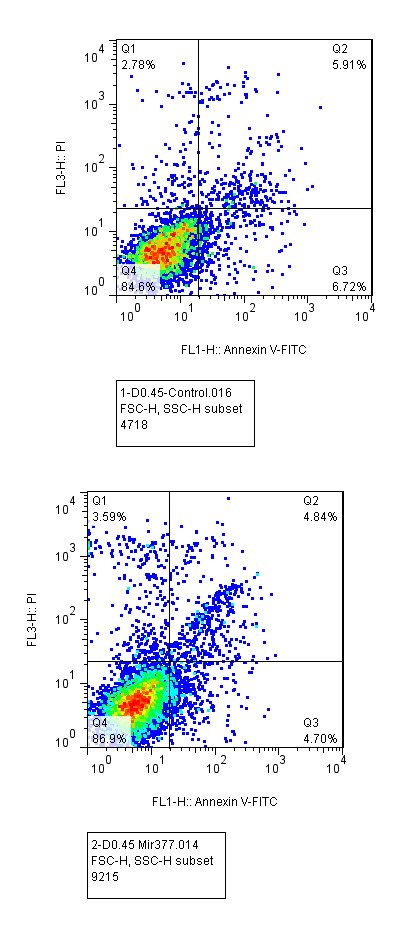

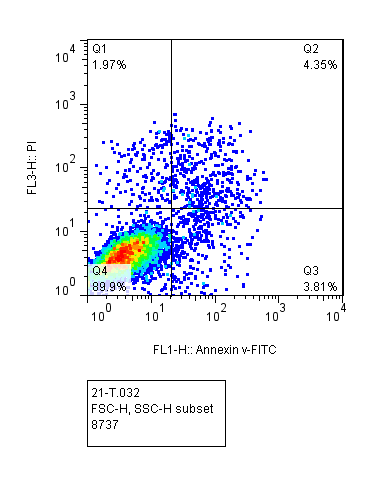

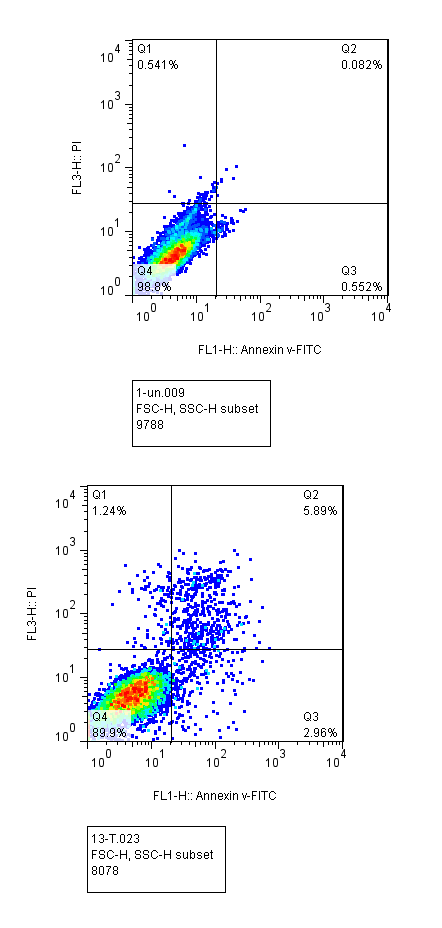

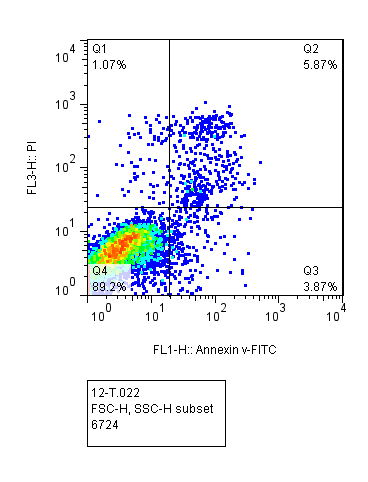

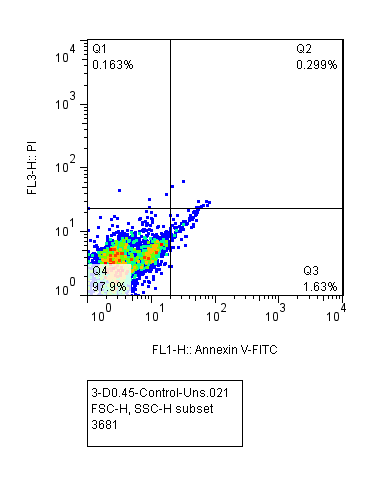


**Control**

**Scrambled**

**miR-335**

**miR-145**


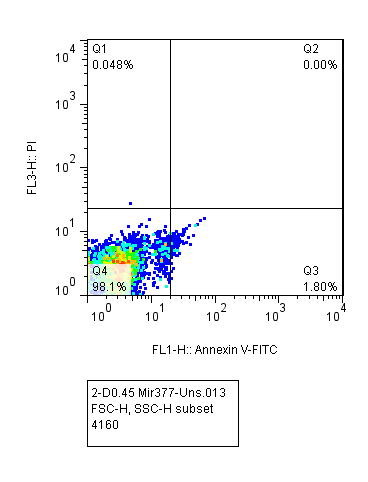


**FL3-H:: PI**

**FL1-H:: Annexin v-FITC**

**BT549**

**MDA-MB231**

**MCF7**

**Q1: Necrosis Q2: Late Apoptosis Q3: Early Apoptosis Q4: Live Cells**


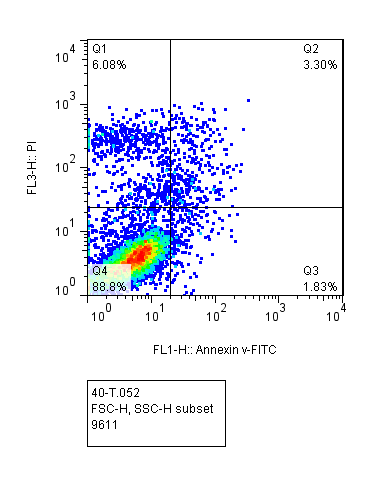


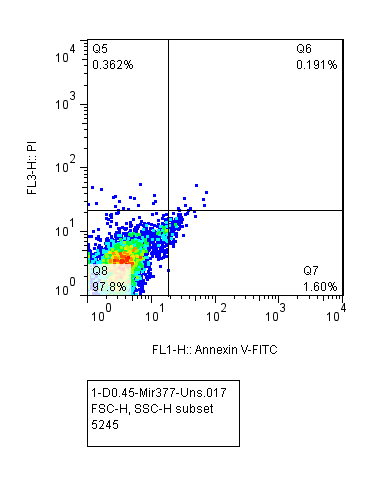


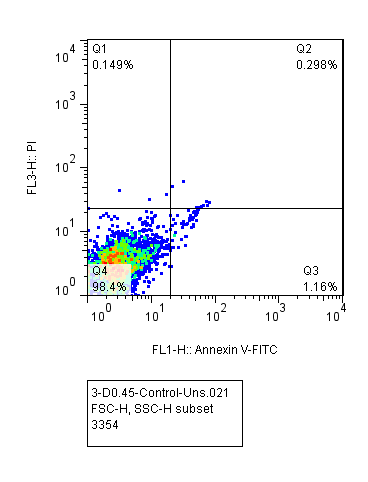


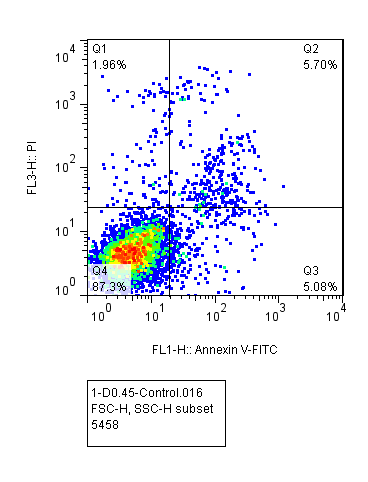


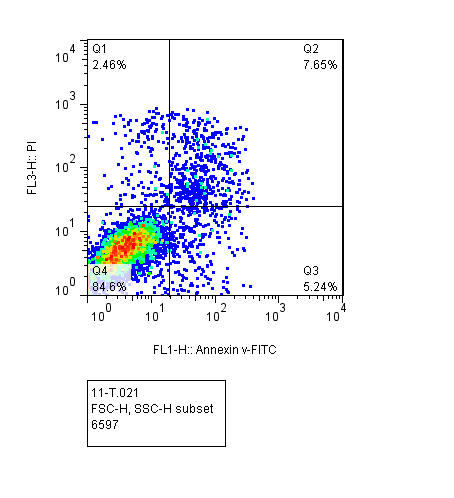


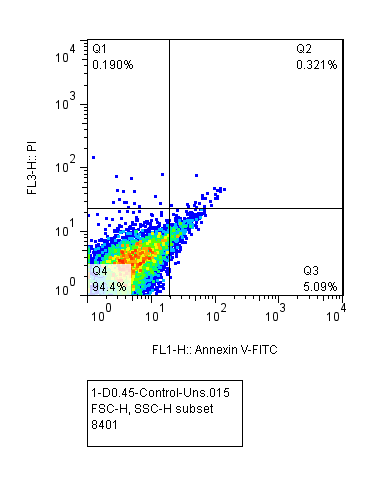

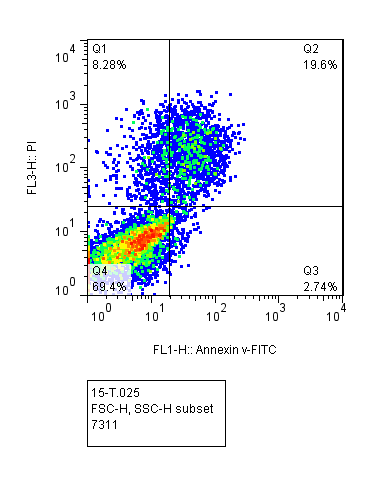

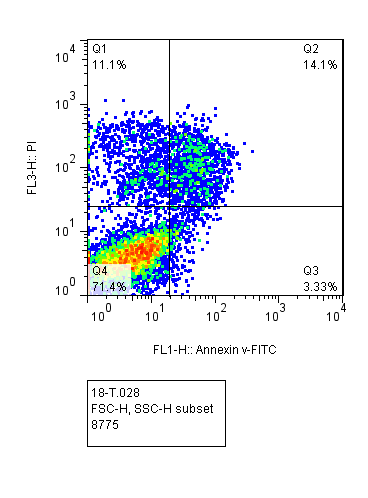

Supplement: Supplementary file 1 — Supplementary Information. [file 41598_2023_27415_MOESM1_ESM.docx]
